# S100A12 as Biomarker of Disease Severity and Prognosis in Patients With Idiopathic Pulmonary Fibrosis

**DOI:** 10.3389/fimmu.2022.810338

**Published:** 2022-02-04

**Authors:** Yupeng Li, Yaowu He, Shibin Chen, Qi Wang, Yi Yang, Danting Shen, Jing Ma, Zhe Wen, Shangwei Ning, Hong Chen

**Affiliations:** ^1^Department of Pulmonary and Critical Care Medicine, Second Affiliated Hospital of Harbin Medical University, Harbin, China; ^2^Medical Research Center, Beijing Chao-Yang Hospital, Capital Medical University, Beijing, China; ^3^College of Bioinformatics Science and Technology, Harbin Medical University, Harbin, China

**Keywords:** Idiopathic pulmonary fibrosis, biomarker, S100A12, prognosis, inflammation

## Abstract

**Background:**

Idiopathic pulmonary fibrosis (IPF) is one of interstitial lung diseases (ILDs) with poor prognosis. S100 calcium binding protein A12 (S100A12) has been reported as a prognostic serum biomarker in the IPF, but its correlation with IPF remains unclear in the lung tissue and bronchoalveolar lavage fluids (BALF).

**Methods:**

Datasets were collected from the Gene Expression Omnibus (GEO) database. Person correlation coefficient, Kaplan–Meier analysis, Cox regression analysis, functional enrichment analysis and so on were used. And single cell RNA-sequencing (scRNA-seq) analysis was also used to explore the role of S100A12 and related genes in the IPF.

**Results:**

S100A12 was mainly and highly expressed in the monocytes, and its expression was downregulated in the lung of patients with IPF according to scRNA-seq and the transcriptome analysis. However, S100A12 expression was upregulated both in blood and BALF of patients with IPF. In addition, 10 genes were found to interact with S100A12 according to protein–protein interaction (PPI) network, and the first four transcription factors (TF) targeted these genes were found according to hTFtarget database. Two most significant co-expression genes of S100A12 were S100A8 and S100A9. The 3 genes were significantly negatively associated with lung function and positively associated with the St. George’s Respiratory Questionnaire (SGRQ) scores in the lung of patients with IPF. And, high expression of the 3 genes was associated with higher mortality in the BALF, and shorter transplant-free survival (TFS) and progression-free survival (PFS) time in the blood. Prognostic predictive value of S100A12 was more superior to S100A8 and S100A9 in patients with IPF, and the composited variable [S100A12 + GAP index (gender, age, and physiological index)] may be a more effective predictive index.

**Conclusion:**

These results imply that S100A12 might be an efficient disease severity and prognostic biomarker in patients with IPF.

## Introduction

Idiopathic pulmonary fibrosis (IPF) is a chronic, progressive, and fibrotic interstitial pneumonia of unknown etiology with repeated acute lung injury, leading to worsening dyspnea and deteriorating lung function (1). The prognosis of patients with IPF is poor usually dying within 2–3 years after diagnosis (2, 3), and the 5-year survival rate is less than 40% (4, 5). Therefore, it is important to identify effective biomarkers for the early identification of patients with a worse prognosis.

Studies had shown that congenital and adaptive immune processes could coordinate existing fibrosis responses (6, 7), and elevated monocyte count of blood were associated with increased risks of IPF progression, hospitalization, and mortality (8, 9). S100A12 (a member of the S100 family of calcium-binding proteins) were mainly and highly expressed in the monocyte cluster according to single cell RNA-sequencing (scRNA-seq) analysis of lung tissue in patients with IPF (10–12). S100A12 takes an important role in the adhesion and migration of leukocytes, and production of cytokines and chemokines according to UniProt database (13). S100A12 could stimulate innate immune cells by binding to advanced glycosylated end product receptor (AGER) (14). In addition, Kang et al. found that S100A12 could activate airway epithelial cells to produce MUC5AC (mucin 5AC, oligomeric mucus/gel-forming) (15). Also, S100A12 could inhibit lung fibroblast migration according to RAGE-p38 MAPK (mitogen-activated protein kinase) signaling (16). Previous studies had demonstrated that S100A12 was upregulated in the serum of patients with IPF, and high expression of S100A12 was associated with higher mortality in patients with IPF (17, 18). However, the correlation between S100A12 and lung function, and the role of S100A12 in the lung tissue and BALF of patients with IPF are unclear.

Therefore, in this study, we used publicly available datasets in the Gene Expression Omnibus (GEO) database to evaluate the association between S100A12 and lung function, and the role of S100A12 in patients with IPF.

## Materials and Methods

### Dataset Preprocessing

**Figure 1:**
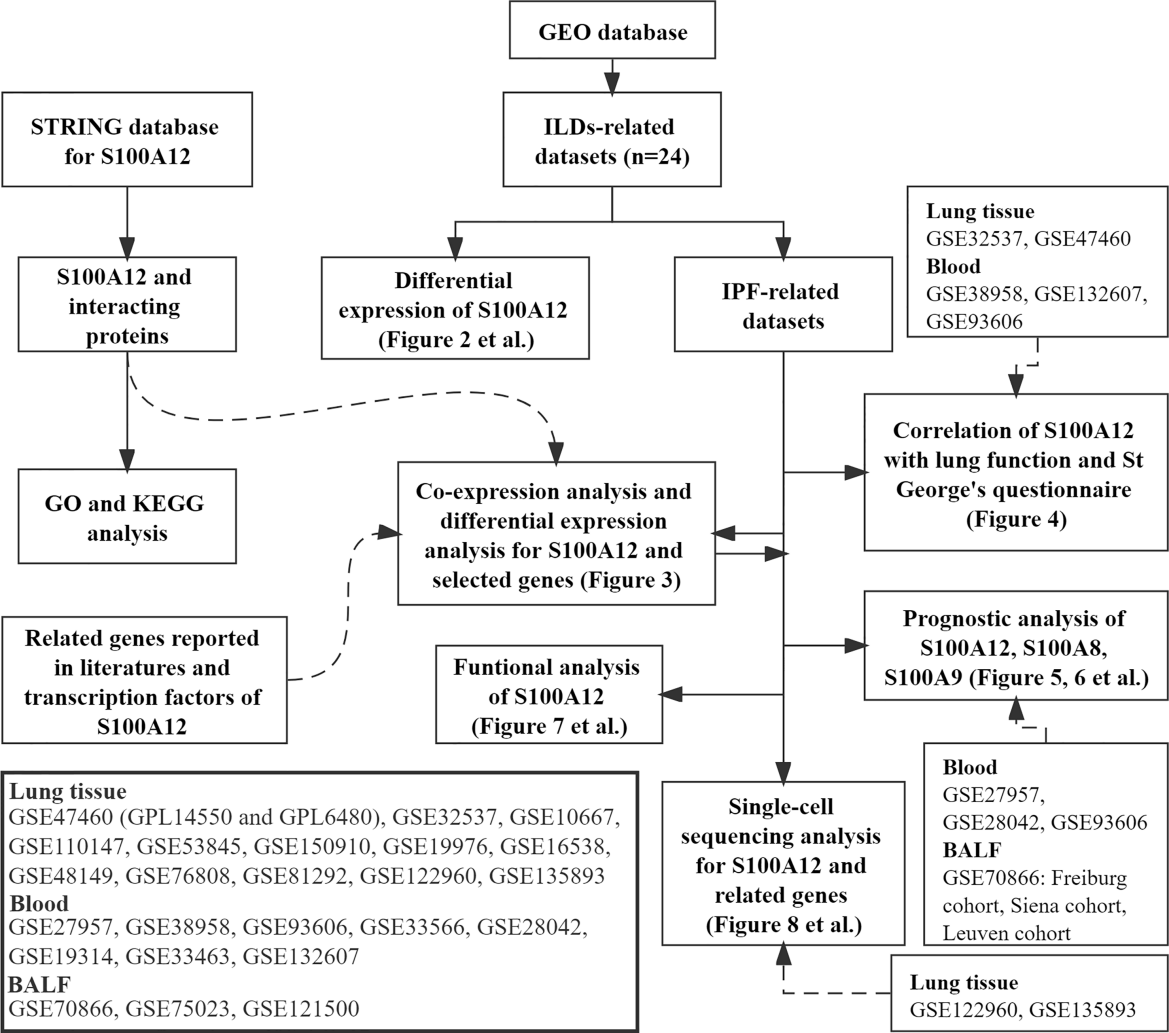
Workflow of this study.

**Figure 1** shows the workflow of our study. According to the GEO database (http://www.ncbi.nlm.nih.gov/geo/), 24 datasets were selected: 13 datasets came from lung tissue samples [GSE47460 (Agilent) (19), GSE32537 (Affymetrix) (20), GSE10667 (Agilent) (21), GSE110147 (Affymetrix) (22), GSE53845 (Agilent) (23), GSE150910 (Illumina) (24), GSE19976 (Affymetrix) (25), GSE16538 (Affymetrix) (26), GSE48149 (Illumina) (27), GSE76808 (Affymetrix) (28), GSE81292 (Affymetrix) (29), GSE122960 (Illumina) (10), and GSE135893 (Illumina) (11)]; 8 datasets came from blood samples [GSE28042 (Agilent) (18), GSE93606 (Affymetrix) (30), GSE33463 (Illumina) (31), GSE19314 (Affymetrix) (32), GSE33566 (Agilent) (33), GSE132607 (Affymetrix) (34), GSE27957 (Affymetrix) (18), and GSE38958 (Affymetrix) (35)]; 3 datasets came from bronchoalveolar lavage fluids (BALF) samples [GSE70866 (Agilent) (36), GSE75023 (Affymetrix) (37), and GSE121500 (Affymetrix) (38)]. R package “Affy” (39) was used to normalize the array data. Approval of the Ethics Committee was not required because the information of patients was obtained from the GEO.

The clinical features of each dataset were showed in the **Table S1**. Percent predicted forced vital capacity (FVC% predicted) and percent predicted diffusion capacity of the lung for carbon monoxide (Dlco% predicted) were extracted from the GSE38958, GSE93606, and GSE132607 datasets (blood). FVC% predicted, Dlco% predicted, and the St. George’s Respiratory Questionnaire (SGRQ**)** scores were extracted from the GSE32537 dataset (lung). FVC% predicted, Dlco% predicted, and percent predicted forced expiratory volume in the first second (FEV1% predicted) were extracted from the GSE47460 dataset (lung). Lung function data in the GSE33566 dataset are incomplete. Therefore, lung function data of this dataset were not used in this study. Transplant-free survival (TFS) was extracted from GSE27957 and GSE28042 datasets (blood). For progression-free survival (PFS), patients with IPF were followed up from the blood draw until (1) disease progression, defined as the decline in FVC% predicted >10% over six months period; (2) death; or (3) censoring at the last contact. Progression-free survival (PFS) was extracted from GSE93606, and GAP index (gender, age, and physiological index) was calculated according to previous report (40) in the GSE93606 dataset (blood). Furthermore, follow-up transcriptome data (1, 3, 6, 12 months) were also extracted from the GSE93606 datasets. GSE70866 dataset was consisted of 3 cohorts (FREIBURG, SIENA, and LEUVEN), and survival and GAP data were extracted from this dataset.

R package “stats” (v.4.0.5, Spearman correlation analysis) was used to determine the association between S100A12 and other genes or lung function among these datasets. Heatmap was constructed by the R packages “gplots” (v.3.1.1), “pheatmap” (v.1.0.12) and “RColorBrewer (v.1.1-2)”. Forest plot was constructed by R package “forestplot” (v.1.10.1, https://CRAN.R-project.org/package=forestplot).

### The Identification of S100A12-Related Partners and Transcription Factors (TFs)

Protein–protein interaction (PPI) network was constructed based on the STRING database (http://www.string.embl.de/, version: 11.0b) (41), and was visualized according to Cytoscape (a software platform for visualizing complex networks, v3.8.2). MEM database (42) (https://biit.cs.ut.ee/mem/index.cgi) was used to verify the correlation between S100A12 and genes came from STRING based on hundreds of publicly available gene expression datasets. Transcription factors (TFs) are key regulators modulated the expression of target genes. In this study, hTFtarget database was used to find out the TFs targeted both S100A12 and its partners.

### The Prognosis-Related Analysis

Kaplan–Meier analysis with the log-rank test was performed to compare TFS or PFS or survival among different groups according to R package “survival” (v.3.2-7). The optimal cut-off value of genes was determined for the survival analysis according to the “surv_cutpoint” function of the R package “survminer” (https://CRAN.R-project.org/package=survminer, v.0.4.8). Univariate cox regression was used to estimate the hazard ratio (HR) of non-TFS or non-PFS or death. Multivariate Cox regression was used for the combined analysis of genes and other variables. Time‐dependent ROC (receiver operator characteristic) curve was constructed to evaluate the predictive value of variables according to the R package “survivalROC” (https://CRAN.R-project.org/package=survivalROC, v.1.0.3).

### Functional Analysis

Gene Ontology (GO) and Kyoto Encyclopedia of Genes and Genomes (KEGG) based on the DEGs [|log Fold Change (logFC)| >1 and false discovery rates (FDR) <0.05] between the patients with high-expression S100A12 and low-expression S100A12 were analyzed and visualized by R package “clusterProfiler” (v.3.18.1) (43). P-values were adjusted with the Benjamini–Hochberg (BH) method. R package “Limma” package (v.3.46.0) (44) was used for the analysis of DEGs. In addition, gene set enrichment analysis (GSEA) method of R package “clusterProfiler” (v.3.18.1) was carried out for the KEGG analysis of all genes. Single-sample gene set enrichment analysis (ssGSEA) was used to calculate the infiltrating score of 19 immune cells and the activity of 15 immune-related pathways (**Table S2**) (45) according to the “GSVA” R package (v.1.38.2) (46). CIBERSORT (47) is a useful analysis tool of RNA mixtures for cellular biomarkers based on the gene expression feature sets of 22 immune cell subtypes (http://cibersort.stanford.edu/). Sound code downloaded from the official website of CIBERSORT was used to calculate the 22 immune cell subtypes score in patients with IPF. Subsequently, the 22 immune cell subtypes were classified into four types: lymphocytes, macrophages, dendritic cell, and mast cell as previously described (48).

### Analysis of scRNA-seq Data

The computational analysis of the GSE122960 and GSE135893 dataset (lung) was performed using R package “Seurat” (4.0.3) (49). Quality control (200 <number of feature RNA <5,000, percentage of mitochondrial genes <20%, percentage of ribosomal genes >3, and percentage of erythrocyte gene <0.1) was respectively performed in the two datasets according to R package “Seurat”. Principal component analysis (PCA) was calculated using the Seurat RunPCA () function. Seurat NormalizeData () function was used to normalize the scRNA-seq data. Seurat FindIntegrationAnchors () and IntegrateData () function based on robust principal component analysis (RPCA) were used to integrate multiple samples. UMAP (uniform manifold approximation and projection) for dimension reduction and Louvain clusters were calculated using the first 30 principal components with the Seurat RunUMAP () and FindClusters () functions, respectively. Resolution was set as 0.8. Seurat FindAllMarkers () function was used to find markers of clusters, and cell types were identified based on markers of each cluster according to CellMarker (50), PanglaoDB databases (51), and the original articles of the two datasets. Expression and distribution of genes were visualized according to Seurat DotPlot (), VlnPlot () and FeaturePlot () functions. R package “Limma” package (v.3.46.0) was used for the analysis of DEGs (logFC >0.25, average expression >1, and false discovery rates (FDR) <0.05) between patients with IPF and control participants in the monocytes with S100A12 >0.

### Statistical Analysis

SPSS Statistics 23 (IBM SPSS) and R software (Version 4.1.0) were used for statistical analysis. Continuous variables were compared by Mann–Whitney U tests. Some statistical analyses were visualized by GraphPad Prism 9. Bilateral test was used.

## Results

### S100A12 Expression in Patients With Interstitial Lung Diseases (ILDs)

**Figure 2:**
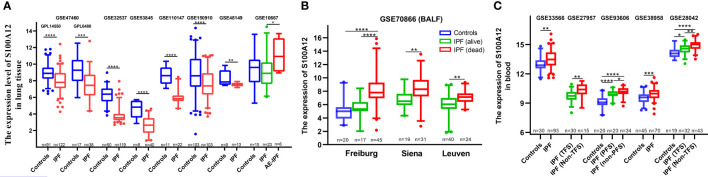
Expression of S100A12 of lung tissue, BALF and blood in patients with IPF. **(A)** The expression of S100A12 in human lung. **(B)** The expression of S100A12 in the BALF. **(C)** The expression of S100A12 in the blood. P-values were showed as: *P < 0.05; **P < 0.01; ***P < 0.001; ****P < 0.0001.

S100A12 is mainly expressed in the bone marrow of human according to the National Center of Biotechnology Information database (**Supplementary Figure 1A**, https://www.ncbi.nlm.nih.gov/gene/6283). RNA sequencing (RNA-seq) and proteomic data of normal subjects showed that S100A12 was highly expressed in immune cells and interstitial cells in adult lung according to lungMAP database (52), (**Supplementary Figures 1B, C**). In order to explore the expression of S100A12 in ILDs, 21 datasets were extracted from the GEO database. The results showed a significant downregulation of lung S100A12 expression in patients with IPF, while S100A12 expression of blood and BALF samples were significantly upregulated especially in patients with poor prognosis (**Figure 2**). In addition, lung S100A12 was significantly downregulated in patients with non-specific interstitial pneumonia (NSIP), systemic sclerosis-related ILD (SSc-ILD), and respiratory bronchiolitis-related ILD (RB-ILD) compared with control participants (**Supplementary Figures 2A–D**). However, the significant difference of S100A12 was not well found in the patients with sarcoidosis (**Supplementary Figure 2E**).

### Partners and TFs of S100A12

**Figure 3:**
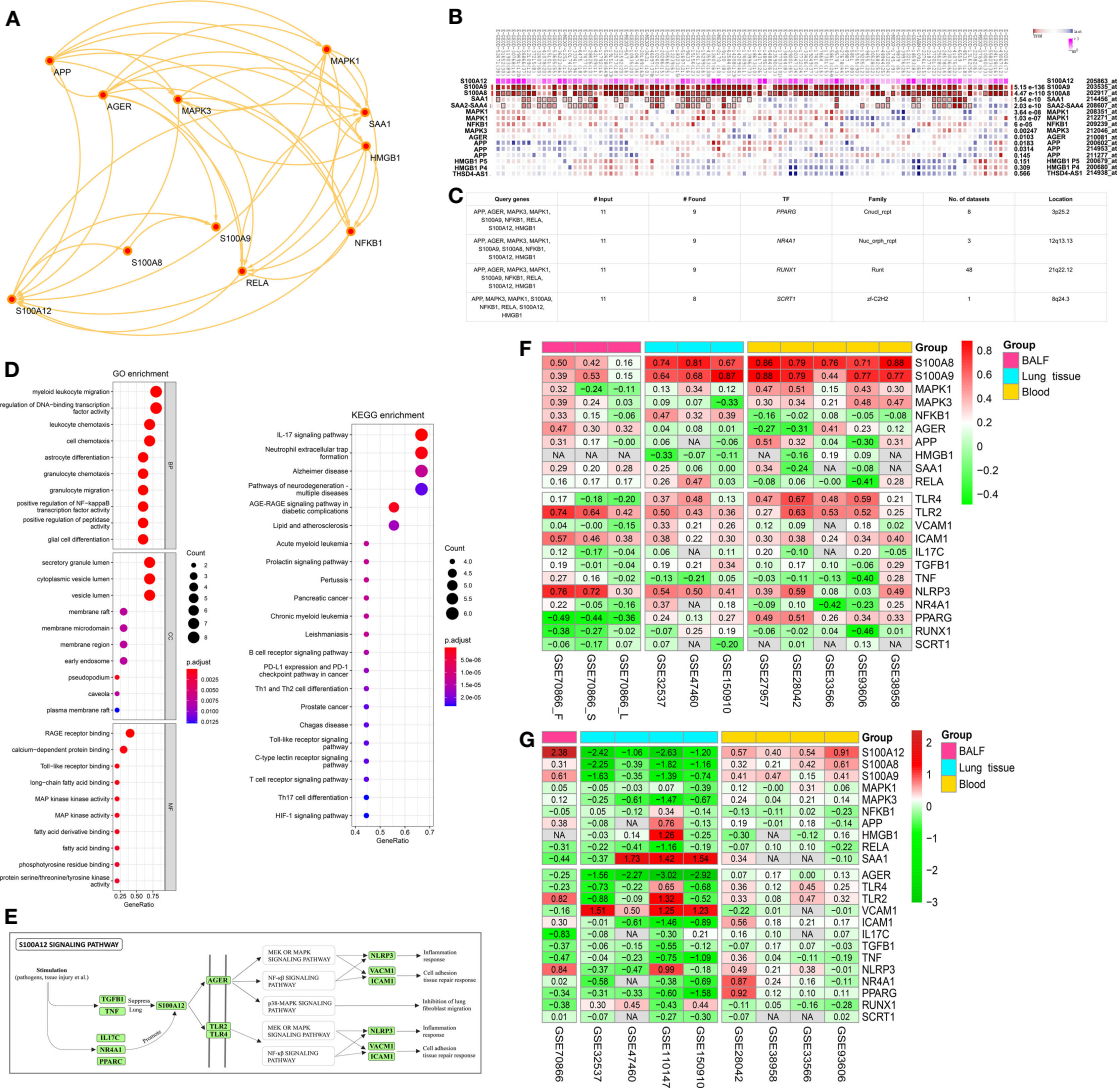
S100A12-related partners and TFs. **(A)** A protein–protein interaction (PPI) network of S100A12 according to STRING database. **(B)** The correlation between S100A12 and the 10 genes came from the PPI network according to MEM database. **(C)** According to the hTFtarget database, the first four TFs of both S100A12 and the 10 interacted genes were found. **(D)** GO and KEGG pathway analysis of both S100A12 and the 10 interacted genes. **(E)** Potential S100A12 signaling pathway based on the literatures of PubMed. **(F)** Co-expression analysis between S100A12 and above genes according to Spearman correlation analysis. Red represents positive correlation, and green represents negative correlation. The numbers represent the correlation coefficients, and the darker color represents the better correlation. **(G)** Heatmap of differential expression analysis between IPF patients and controls according to R package “Limma”. Red represents upregulation, and green represents downregulation. The numbers represent the logFC, and the darker color represents the more notable difference. NA, not available.

According to the STRING database, 10 protein coding genes (S100A8, S100A9, RELA, MAPK3, AGER, APP, NFKB1, HMGB1, SAA1, and MAPK1) were found to interact with S100A12 (**Figure 3A**). Based on MEM database, S100A8 and S100A9 were the most notable co-expression genes of S100A12 according to hundreds of datasets **(****Figure 3B**). According to the hTFtarget database, the first four TFs (PPARG, NR4A1, RUNX1, and SCRT1) of both S100A12 and the 10 interacted genes were selected for further study (**Figure 3C**). S100A12 and the 10 genes were significantly associated with NF-kappaB transcription factor activity, RAGE receptor binding, MAP kinase activity, the IL-17 signaling pathway, B cell receptor signaling pathway, and T cell receptor signaling pathway according to GO and KEGG analysis (**Figure 3D**). According to reviewing the literatures published on the PubMed, a potential mechanism of S100A12 was summarized in the **Figure 3E**. And, the potential up-stream and down-stream genes were also showed.

### Co-Expression Analysis and Differential Expression Analysis

Spearman correlation analysis between S100A12 and the above genes was conducted in patients with IPF (**Figure 3F**). In BALF, 7 genes (S100A8/9, AGER, SAA1, TLR2, ICAM1, and NLRP3) were positively associated with S100A12, and PPARG was negatively associated with S100A12. In the lung, 11 genes (S100A8/9, NFKB1, TLR4, TLR2, VCAM1, ICAM1, TGFB1, NLRP3, NR4A1, PPARG) were positively associated with S100A12. In the blood, 9 genes (S100A8/9, MAPK1/3, TLR4/2, ICAM1, NLRP3, and PPARG) were positively associated with S100A12. These results showed that S100A8/9, TLR2, ICAM1, and NLRP3 were the most notable genes positively associated with S100A12 among the 3 tissues. Furthermore, differential expression analysis was conducted between patients with IPF and controls according to R package “Limma” (**Figure 3G**). In the BALF, S100A12, S100A9, TLR2, and NLRP3 were significantly upregulated, whereas, IL17C, TNF, and RUNX1 were significantly downregulated. In the lung, S100A12, S100A8/9, MAPK3, AGER, ICAM1, and PPARG were significantly downregulated, whereas, VCAM1 were significantly upregulated. In the blood, S100A12 and S100A8/9 were significantly upregulated. These results showed that S100A8 and S100A9 were the most co-expression genes associated with S100A12, which were included for further study.

### Correlation Between Genes and Lung Function or SGRQ Scores

**Figure 4:**
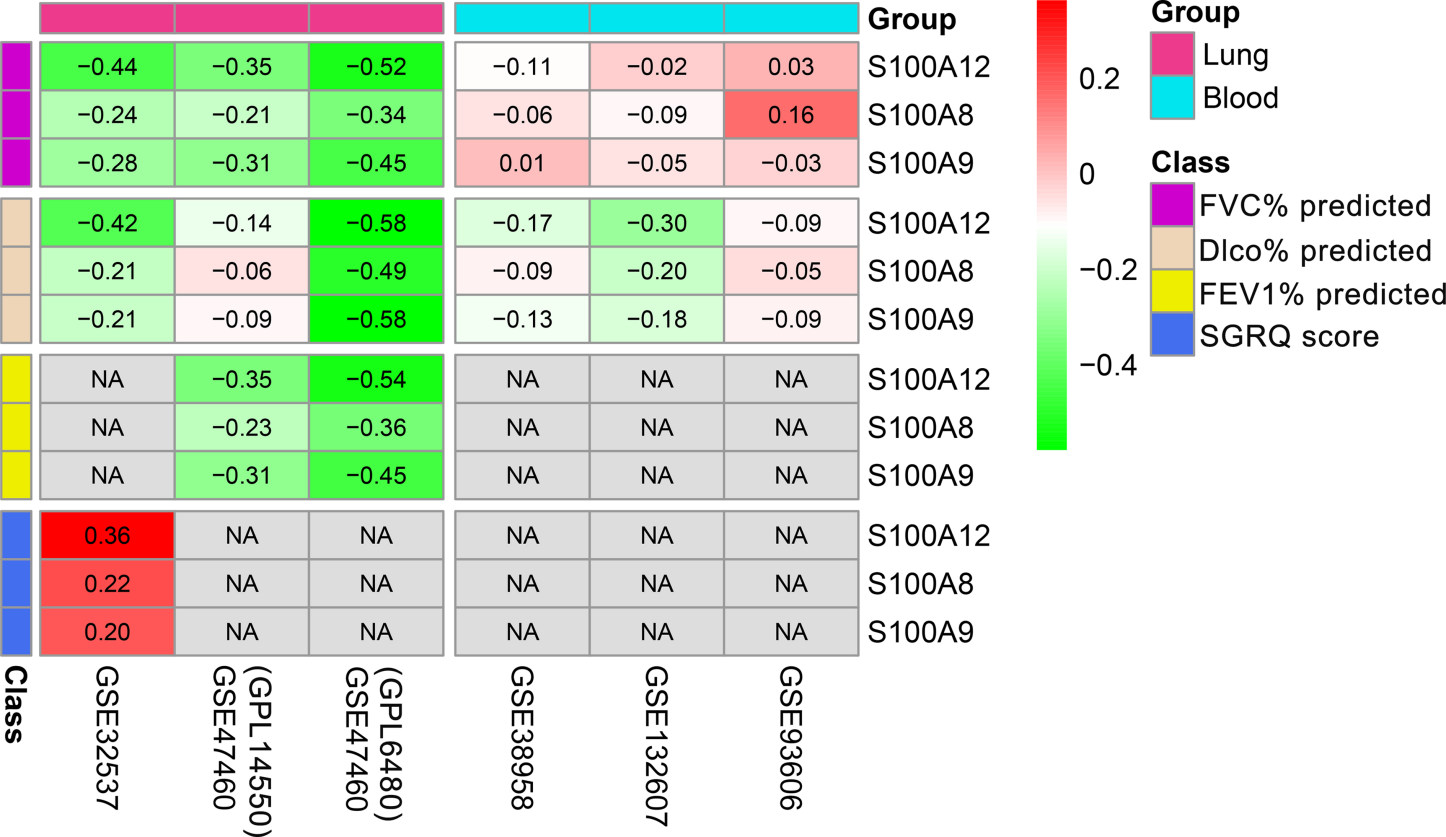
Correlation between 3 genes (S100A12, S100A8, and S100A9) and lung function or SGRQ score in the lung and blood of patients with IPF. NA, not available.

S100A12, S100A8, and S100A9 were significantly negatively associated with FVC% predicted and Dlco% predicted, and were significantly positively associated with SGRQ scores in the GSE32537 dataset (lung tissue, **Figure 4**). GSE47460 dataset (lung tissue) consisted of two platforms: GPL14550 and GPL6480. The two cohorts confirmed the significant negative association between S100A12 and lung function, and found that the 3 genes were also significantly negatively associated with FEV1% predicted (**Figure 4**). However, there was no significant correlation between S100A12 and lung function in the blood datasets (**Figure 4**). In addition, S100A12 had more significant correlation compared with S100A8 and S100A9.

### Prognosis-Related Analysis

**Figure 5:**
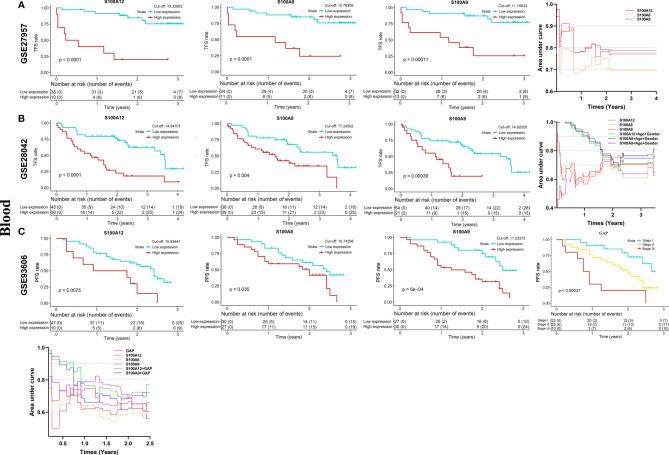
K-M analysis and the predictive value of S100A12, S100A8, S100A9 and GAP index for the TFS and PFS in the blood of patients with IPF. **(A)** GSE27957 dataset (TFS), **(B)** GSE28042 dataset (TFS), **(C)** GSE93606 dataset (PFS). The acquisition of composite variable such as S100A12 + GAP was based on the multivariable Cox regression.

**Figure 6:**
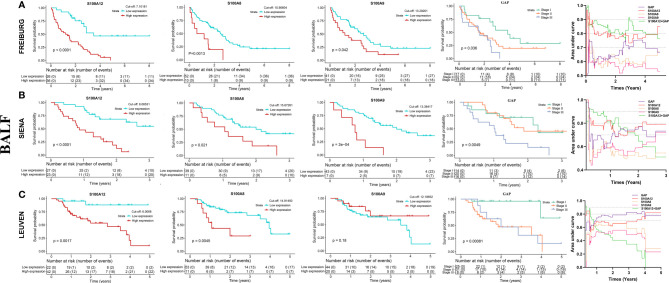
K–M analysis and the predictive value of S100A12, S100A8, S100A9 and GAP index for the survival in the BALF of patients with IPF according to GSE70866 dataset. **(A)** FREIBURG cohort, **(B)** SIENA cohort, **(C)** LEUVEN cohort. The acquisition of composite variable such as S100A12 + GAP was based on the multivariable Cox regression.

Patients with IPF were divided into two groups based on the optimal cut-off value of genes as described in the methods section. In the blood (GSE27957 and GSE28042), patients with high-expressions S100A12 or S100A8 or S100A9 were significantly associated with shorter TFS (transplant-free survival) time than those with low expression (**Figures 5A, B**, **Supplementary Figure 3A**). The areas under curve (AUCs) showed that the predicted value of S100A12 for TFS was slightly higher than S100A8 and S100A9 (**Table S3** and **Figures 5A, B**). Because the dead patients are all male in the GSE27957 dataset (blood), the result of composited prognostic index might be difficult to assess (**Supplementary Table 3****)**. Based on the GSE28042 dataset (blood), the predictive values of models (S100A8/9/12 + age + gender) were superior to the single index (**Table S3** and **Figure 5B**). In addition, S100A12, S100A8, S100A9, and GAP index (gender, age, and physiological index) were significantly negatively associated with PFS (progression-free survival) in the GSE93606 dataset (blood, **Figure 5C** and **Supplementary Figure 3B**). According to AUC, the model consisted of S100A12 and GAP had better predictive value compared with that consisted of S100A9 and GAP (**Table S3** and **Figure 5C**). In the BALF, S100A12 had better predictive value for mortality compared with S100A8, S100A9, and GAP (**Supplementary Figure 4**, **Figures 6A–C**). Also, the model consisting of S100A12 and GAP was considered as the more effective predictive model for the mortality according to ROC curve analysis (**Table S3**, **Figures 6A–C**).

### Functional Analysis

In order to reveal the underlying biological functions and pathways correlated with S100A12, GO enrichment and KEGG pathway analysis of DEGs [|logFC| >1 and FDR <0.05] between patients with high-expression and low-expression S100A12 were performed. In the 3 tissues of patients with IPF, the significant GO terms and KEGG pathways were mainly enriched in neutrophil activation, immune receptor activity, regulation of inflammatory response, RAGE receptor binding, IL-17 signaling pathway, cytokine-cytokine receptor interaction, TNF signaling pathway, PI3K-Akt signaling pathway, NOD-like receptor signaling pathway, and chemokine signaling pathway (**Supplementary Figures 5**–**7**). These results were consistent with the GSEA analysis (**Supplementary Figure 8**).

**Figure 7:**
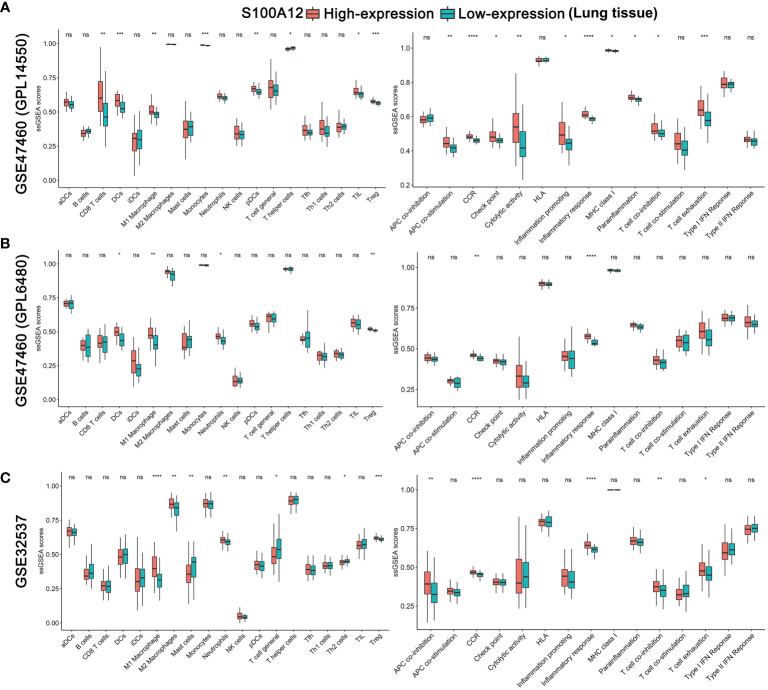
Comparison of the lung ssGSEA scores between patients with high-expression and low-expression S100A12 in the GSE47460 (GPL14550) **(A)**, GSE47460 (GPL6480) **(B)**, and GSE32537 datasets **(C)**. The scores of 19 immune cells are displayed in the left side, and 15 immune-related functions are displayed in the right side. DC, Dendritic Cell; TIL, Tumor infiltrates lymphocytes; CCR, cytokine-cytokine receptor. P-values were showed as: ns, not significant; *P < 0.05; **P < 0.01; ***P < 0.001; ****P < 0.0001.

In addition, ssGSEA analysis showed that patients with high-expression S100A12 were more likely to have higher scores of dendritic cells (DCs), M1 macrophages, neutrophils, regulatory T cells (Treg), cytokine-cytokine receptor (CCR), inflammatory response, and T cell exhaustion compared with patients with low-expression S100A12 in the lung (**Figure 7**). In the BALF, high expression of S100A12 was also significantly associated with higher score of inflammatory response (**Supplementary Figure 9**). Interestingly, in the blood, patients with high-expression S100A12 were more likely to have lower scores of B cells, CD8 T cells, Th1 cells, Th2 cells, tumor infiltrates lymphocytes (TIL), and check point (**Supplementary Figure 10**). These results revealed that S100A12 was significantly positively associated with the inflammatory process, and the high expression of S100A12 might be associated with lower immune activity. Furthermore, CIBERSORT analysis verified that S100A12 was significantly positively associated with the inflammatory process in the 3 tissues (**Supplementary Figures 11**–**13**).

### The scRNA-seq Analysis

**Figure 8:**
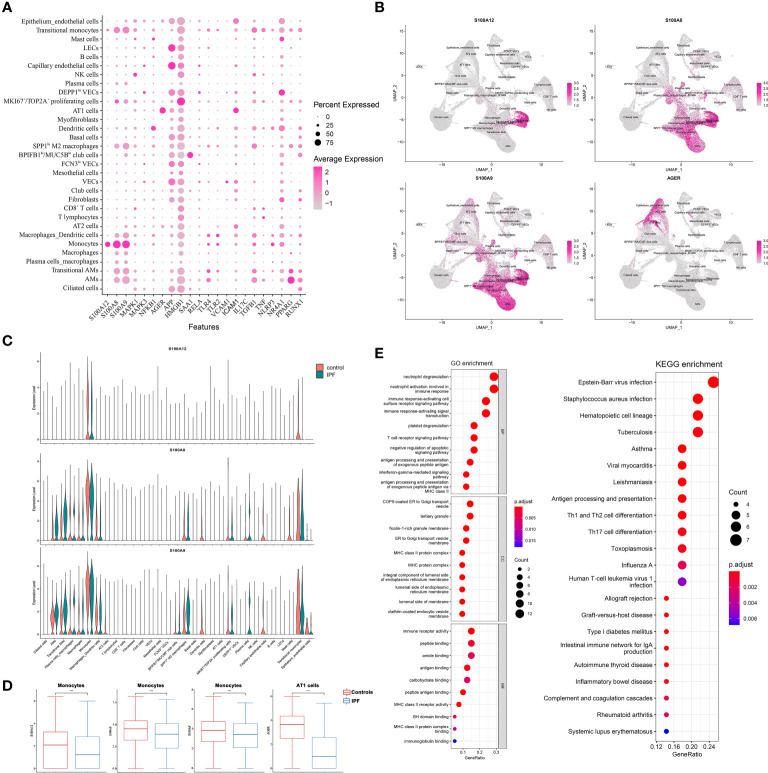
The scRNA-seq analysis in the GSE135893 dataset (IPF = 12, control = 10). **(A)** Color dot plot of S100A12 and its partners; **(B)** Feature plot of S100A12, S100A8, S100A9, and AGER; **(C)** Violin plot of S100A12, S100A8, and S100A9. **(D)** The different expressive analysis of S100A12, S100A8, S100A9, and AGER between IPF and controls in the selected cell type; **(E)** The GO and KEGG analysis of DEGs between IPF and controls in the monocytes with S100A12 >0.

Two scRNA-seq datasets [GSE135893 (IPF = 12, control = 10) and GSE122960 (IPF = 4, control = 4)] were selected. S100A12 and AGER were mainly and highly expressed in the monocytes and alveolar epithelial type 1 cells (AT1 cells), respectively (**Figures 8A–C** and **Supplementary Figures 14A–C**). S100A8 and S100A9 were mainly expressed in the monocytes and macrophages (**Figures 8A–C** and **Supplementary Figures 14A–C**). NFKB1 was mainly expressed in the dendritic cells, SAA1 was mainly expressed in the BPIFB1^+^/MUC5B^+^ club cells, and PPARG was mainly expressed in the alveolar macrophages (AMs) (**Figure 8A**). In addition, S100A12, S100A8, and S100A9 were significantly downregulated in the monocytes of patients with IPF, and AGER was also significantly downregulated in the AT1 cells (**Figure 8D** and **Supplementary Figure 14D**). Interestingly, S100A8 and S100A9 were upregulated in the macrophages of IPF. According to the GO and KEGG analysis for the DEGs (**Table S4**) between patients with IPF and controls in the monocytes with S100A12 >0, monocytes with S100A12 >0 of patients with IPF might be more associated with antigen processing and presentation (**Figure 8E** and **Supplementary Figure 14E**). Interestingly, a transitional status of monocytes was observed in the GSE135893 dataset, and expression of S100A12 of transitional monocytes was downregulated in the patients with IPF (**Figure 8C**).

## Discussion

IPF characterized by a radiographic and pathologic pattern of usual interstitial pneumonia (UIP) is a chronic and fatal lung disease. In this study, we found that S100A12 and its 2 most significant co-expression genes (S100A8 and S100A9) were significantly downregulated in the lung of patients with IPF, whereas, they were significantly upregulated in the BALF and blood. Also, they were mainly expressed by the monocytes, and were also significantly downregulated in the monocytes of patients with IPF compared with controls according to scRNA-seq analysis. In addition, according to the function-related bioinformatic analysis (GO, KEGG, GSEA, CIBERSORT analysis, and ssGSEA analysis), patients with high-expression S100A12 were more likely to have higher inflammatory response compared with those with low-expression S100A12. These results were consistent with the functional annotation of UniProt database regarding S100A12 (13).

S100A12 could stimulate immune cells according to binding to AGER (14), and activate the MAPK (mitogen-activated protein kinase) and NF-kappa-B signaling pathways leading to production of proinflammatory cytokines and upregulation of cell adhesion molecules ICAM1 and VCAM1 according to binding to AGER (13, 14). Realegeno and his colleagues also suggested that S100A12 was also associated with toll-like receptor 2 (TLR2) (53). According to the scRNA-seq analysis, AGER was mainly expressed in the AT1 cells, and was significantly downregulated in patients with IPF, which was consistent with the previous reports in the ILDGDB database (54, 55). Furthermore, the loss of AGER in the pulmonary fibrosis was induced by TGFB1 and TNF-alpha (54). Studies suggested that S100A12 could inhibit lung fibroblast migration according to RAGE-p38 MAPK signaling (16, 56–58). Based on the scRNA-seq analysis, the analysis for monocytes with S100A12 expression showed that monocytes of patients with IPF were more likely to have higher functions of DCs compared with those of control participants, and a transitional status was found, which imply the differentiated ability of monocytes in the IPF. In the process of pulmonary fibrosis, monocytes are recruited into the lung in response to tissue injury and differentiate into long-lived macrophages producing TGF-β, CCL18, CHI3L1, MMPs, eventually, leading to fibroblast activation, myofibroblast differentiation, and extracellular matrix (ECM) remodeling (7). Studies had found that mRNA and protein level of S100A12 were significantly decreased during monocyte-to-macrophage differentiation (59, 60). Therefore, we speculated that the expression of S100A12 may be similarly inhibited by TGFB1 and TNF-alpha in the process of fibrosis, or low expression of S100A12 may be caused by the differentiation of monocytes, which needs further study to verify. However, there is one important gap for the exploration of mechanism of S100A12: murine S100A12 is absent (61).

Previous studies showed that the heterodimer S100A8/A9 protein were significantly upregulated in the lung of patients with IPF, and the two genes may promote the development of fibrosis (62). However, in this study, the mRNA levels of the two genes were significantly downregulated in lung of patients with IPF compared with controls according to the high-throughput datasets. We speculated that the mRNA level of S100A8 and S100A9 may be not matched with the protein level of them. According to scRNA-seq analysis, S100A12 was expressed exclusively by monocytes, independently from S100A8 and S100A9. S100A8 and S100A9 were expressed by not only monocytes but also macrophages, and their expressions in the macrophages of patients with IPF were higher than controls, which implied that S100A8 and S100A9 may play a role in the development of IPF.

Actually, we were more likely to believe that S100A12 was more likely to reveal the status of host defense of patients with IPF. More and more evidences had shown that the disordered host defense was an important contributor to disease progression in IPF (30, 63, 64). Gastroesophageal reflux disease (GERD) is common in patients with IPF, thereby, continuous micro-aspiration may lead to repeated inoculation with oral and stomach microorganisms, which leads to repetitive alveolar injury and repair (65). Furthermore, studies showed that patients with IPF had higher microbial load compared with normal populations (30, 66). And, higher bacterial load was associated not only with increased risk for disease progression and mortality but also with the presence of s35705950 polymorphism of the MUC5B (mucin 5B, oligomeric mucus/gel-forming), a known predisposing factor for the development of IPF (30, 66). Interestingly, SAA1, one of partners of S100A12, was mainly expressed in the BPIFB1^+^/MUC5B^+^ club cells. S100A12 had been identified as an effective inflammatory biomarker of poor prognosis in the familial Mediterranean fever (67), acute respiratory distress syndrome (ARDS) (68, 69), hemodialysis (70), SSc-ILD (71) and so on. Based on the GO, KEGG, and GSEA analysis, IL17 signaling pathway was the one of important pathways in the patients with high-expression S100A12. As a T helper 17 (Th17) cytokine, IL-17 family was implicated in the pathogenesis of various autoimmune related diseases. IL-17C could enhance the epithelial host defense response by upregulating S100A12 expression (72), moreover, S100A12 could activate airway epithelial cells to produce MUC5AC (10). These results suggested that S100A12 could reveal the status of host defense of patients with IPF well. In this study, compared with alive or progression-free patients with IPF, S100A12 was significantly higher in the BALF and blood of progressive or/and dead patients with IPF. Also, S100A12 was significantly higher in the lung of patients with AE-IPF (acute exacerbation of IPF). Furthermore, high expression of S100A12 was associated not only with increased risk for disease progression and mortality both in the BALF and blood but also with poor lung function and quality of life in the lung. Also, due to the exclusive expression of S100A12 in the monocytes, the prognostic predictive value of S100A12 was more superior to S100A8 and S100A9 in patients with IPF. Additionally, the model consisted of S100A12 and GAP may be more effective than single index, which needs further study to verify.

In a word, based on the current available references, we speculated that the contradictory results of S100A12 between lung and BALF or blood may be caused by several points as follows: (1) S100A12 could inhibit lung fibroblast migration according to RAGE-p38 MAPK signaling. The loss of AGER was caused by TGFB1 and TNF-alpha in the pulmonary fibrosis. Therefore, S100A12 may be inhibited by TGFB1 and TNF-alpha in the development of fibrosis. (2) In the process of pulmonary fibrosis, monocytes are recruited into the lung in response to tissue injury and differentiate into long-lived macrophages. Low expression of S100A12 may be caused by the differentiation of monocytes. (3) S100A12 could reveal the status of host defense of patients with IPF. When the acute exacerbation or high microbial load occurs, S100A12 was upregulated, which may explain that S100A12 was negatively associated with lung function in lung of patients with IPF. (4) S100A12 could activate airway epithelial cells to produce MUC5AC. Mucin takes an important role in the development of IPF. Thereby, S100A12 was upregulated in the BALF of patients with IPF. Further study was necessary to verify the mRNA and protein level of S100A12 in the lung and BALF of patients with IPF.

There are several limitations in this study. First, the study lacked detailed treatment information of patients, which may influence on the predictive value of S100A12. Second, the study was based on the retrospective data from GEO, and the number of samples in each dataset was relatively small. Third, many prominent prognostic clinical parameters such as treatment measures, underlying diseases and so on were not reported in most datasets that we used; thereby, the prognostic value of S100A12 and the correlation between S100A12 and lung function were limited. Finally, larger-sample prospective studies are needed to estimate the clinical relevance of S100A12.

## Conclusion

S100A12 might be an efficient monocyte-specific disease severity and prognostic biomarker in patients with IPF. Also, the composited variable (S100A12 + GAP) may be a more effective predictive index for the prognosis of patients with IPF. In addition, S100A8 and S100A9 were also useful biomarkers for the prediction of poor prognosis. However, further studies are needed to confirm these results and explore the underlying mechanisms.

## Data Availability Statement

The datasets presented in this study can be found in online repositories. The names of the repository/repositories and accession number(s) can be found in the article/**Supplementary Material**.

## Author Contributions

YL developed the research idea, performed data collection, performed data analysis, performed the scRNA-seq analysis, validated data collection, prepared the first manuscript draft, refined the research idea, and edited manuscripts. YH and SC validated data collection, refined the research idea, performed data analysis, prepared the first manuscript draft, and edited manuscripts. QW, YY, DS, JM, and ZW performed data analysis. HC and SN developed the research idea, refined the research idea, validated data collection and edited manuscripts. HC and SN are the guarantors of the manuscript. All authors listed have made a substantial, direct, and intellectual contribution to the work and approved it for publication.

## Funding

This work was supported by grants from the Science and Technology Department, Heilongjiang Province (No. GY2021ZB0198 to HC).

## Conflict of Interest

The authors declare that the research was conducted in the absence of any commercial or financial relationships that could be construed as a potential conflict of interest.

## Publisher’s Note

All claims expressed in this article are solely those of the authors and do not necessarily represent those of their affiliated organizations, or those of the publisher, the editors and the reviewers. Any product that may be evaluated in this article, or claim that may be made by its manufacturer, is not guaranteed or endorsed by the publisher.

## Supplementary Material

Supplementary Figure 1 RNA sequencing data and proteomic profiling of S100A12. **(A)** RNA-seq for S100A12 in different tissues according to National Center of Biotechnology Information database (https://www.ncbi.nlm.nih.gov/gene/6283). RNA-seq analysis **(B)** and proteomic profiling **(C)** for S100A12 in the human lung according to lungMAP database.

Supplementary Figure 2 Expression of S100A12 of lung tissue, blood and BALF. **(A-C)** The expression of S100A12 in human lung. **(D)** The expression of S100A12 in patients with SSc-ILD. **(E)** The expression of S100A12 in patients with sarcoidosis. ^$^P < 0.05 vs. controls; ^#^P < 0.05 vs. patients with COPD. P values were showed as: *P < 0.05; **P < 0.01; ***P < 0.001; ****P < 0.0001. COPD: chronic obstructive pulmonary disease; NSIP: non-specific interstitial pneumonia; HP: hypersensitivity pneumonitis; RB-ILD: respiratory bronchiolitis-related ILD; SSc-ILD: systemic sclerosis-related ILD; PAH: pulmonary arterial hypertension.

Supplementary Figure 3 Results of the cox regression regarding non-TFS in the GSE27957 and GSE28042 datasets **(A)**, and the cox regression regarding non-PFS in the GSE93606 dataset **(B)**.

Supplementary Figure 4 Results of the cox regression regarding mortality in the GSE70866 dataset.

Supplementary Figure 5 Significant GO terms and KEGG pathways of DEGs between patients with high-expression and low-expression S100A12 (lung tissue). The top 10 significant terms for biological processes (BP), cellular component (CC), and molecular function (MF), and significant terms for KEGG pathways in the GSE47460 (GPL14550) **(A)**, GSE47460 (GPL6480) **(B)**, and GSE32537 datasets **(C)**.

Supplementary Figure 6 Significant GO terms and KEGG pathways of DEGs between patients with high-expression and low-expression S100A12 (blood). The top 10 significant terms for biological processes (BP), cellular component (CC), and molecular function (MF), and significant terms for KEGG pathways in the GSE27957 **(A)**, GSE38958 **(B)**, and GSE93606 datasets **(C)**.

Supplementary Figure 7 Significant GO terms and KEGG pathways of DEGs between patients with high-expression and low-expression S100A12 according to GSE70866 dataset (BALF). The top 10 significant terms for biological processes (BP), cellular component (CC), and molecular function (MF), and significant terms for KEGG pathways in the FREIBURG cohort **(A)**, SIENA cohort **(B)**, and LEUVEN cohort **(C)**.

Supplementary Figure 8 GSEA analysis of DEGs between patients with high-expression and low-expression S100A12 in the three tissues. Lung tissue: **(A)** GSE47460 (GPL14550) dataset, **(B)** GSE47460 (GPL6480) dataset, **(C)** GSE32537 dataset. Blood: **(D)** GSE38958 dataset, **(E)** GSE27957 dataset, **(F)** GSE93606 dataset. BALF (GSE70866 dataset): **(G)** FREIBURG cohort, **(H)** SIENA cohort, **(I)** LEUVEN cohort.

Supplementary Figure 9 Comparison of the BALF ssGSEA scores between patients with high-expression and low-expression S100A12 in the FREIBURG cohort **(A)**, SIENA cohort **(B)**, LEUVEN cohort **(C)** in the GSE70866 dataset. The scores of 19 immune cells are displayed in the left side, and 15 immune-related functions are displayed in the right side. DC, Dendritic Cell; TIL, Tumor infiltrates lymphocytes; CCR, cytokine-cytokine receptor. P values were showed as: ns, not significant; *P < 0.05; **P < 0.01; ***P < 0.001; ****P < 0.0001.

Supplementary Figure 10 Comparison of the blood ssGSEA scores between patients with high-expression and low-expression S100A12 in the GSE27957 **(A)**, GSE38958 **(B)**, and GSE93606 datasets **(C)**. The scores of 19 immune cells are displayed in the left side, and 15 immune-related functions are displayed in the right side. DC, Dendritic Cell; TIL, Tumor infiltrates lymphocytes; CCR, cytokine-cytokine receptor. P values were showed as: ns, not significant; *P < 0.05; **P < 0.01; ***P < 0.001; ****P < 0.0001.

Supplementary Figure 11 Comparison of the lung CIBERSORT scores between patients with high-expression and low-expression S100A12 in the GSE47460 (GPL14550) **(A)**, GSE47460 (GPL6480) **(B)**, and GSE32537 datasets **(C)**. The scores of 22 immune cells are displayed in the left side, and the scores of 4 composited cell types are displayed in the right side. P values were showed as: ns, not significant; *P < 0.05; **P < 0.01; ***P < 0.001; ****P < 0.0001.

Supplementary Figure 12 Comparison of the blood CIBERSORT scores between patients with high-expression and low-expression S100A12 in the GSE27957 **(A)**, GSE38958 **(B)**, and GSE93606 datasets **(C)**. The scores of 22 immune cells are displayed in the left side, and the scores of 4 composited cell types are displayed in the right side. P values were showed as: ns, not significant; *P < 0.05; **P < 0.01; ***P < 0.001; ****P < 0.0001.

Supplementary Figure 13 Comparison of the BALF CIBERSORT scores between patients with high-expression and low-expression S100A12 in the FREIBURG cohort **(A)**, SIENA cohort **(B)**, LEUVEN cohort **(C)** in the GSE70866 dataset. The scores of 22 immune cells are displayed in the left side, and the scores of 4 composited cell types are displayed in the right side. P values were showed as: ns, not significant; *P < 0.05; **P < 0.01; ***P < 0.001; ****P < 0.0001.

Supplementary Figure 14 The scRNA-seq analysis in the GSE122960 dataset (IPF = 4, control = 4). **(A)** Color dot plot of S100A12 and its partners; **(B)** Feature plot of S100A12, S100A8, S100A9, and AGER; **(C)** Violin plot of S100A12, S100A8, and S100A9. **(D)** The different expressive analysis of S100A12, S100A8, S100A9, and AGER between IPF controls in the selected cell; **(E)** The GO and KEGG analysis of DEGs between IPF and controls in the monocytes with S100A12 > 0.
