# Supplementary figures and images for: S100A12 as Biomarker of Disease Severity and Prognosis in Patients With Idiopathic Pulmonary Fibrosis
Source: Front Immunol. 2022 Feb 4;13:810338. doi: 10.3389/fimmu.2022.810338 (PMC8854978; doi:10.3389/fimmu.2022.810338)

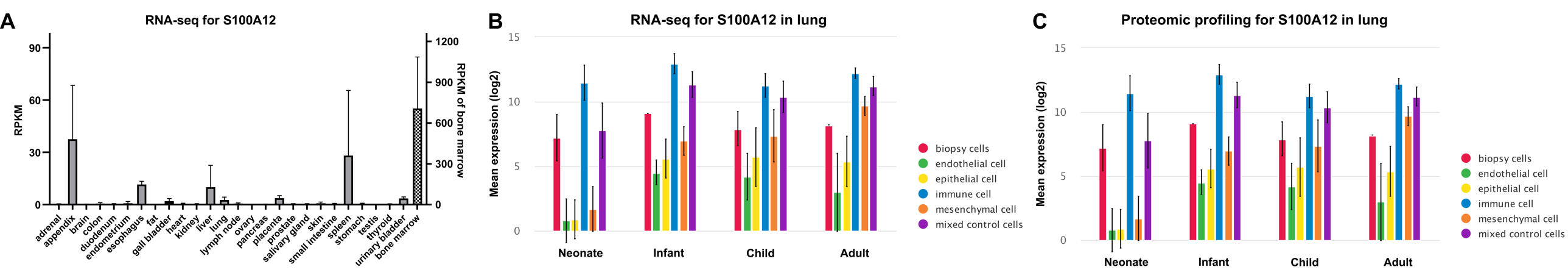

Supplement: Supplementary Figure 1 — RNA sequencing data and proteomic profiling of S100A12. (A) RNA-seq for S100A12 in different tissues according to National Center of Biotechnology Information database (https://www.ncbi.nlm.nih.gov/gene/6283). RNA-seq analysis (B) and proteomic profiling (C) for S100A12 in the human lung according to lungMAP database. [file Image_1.tif]

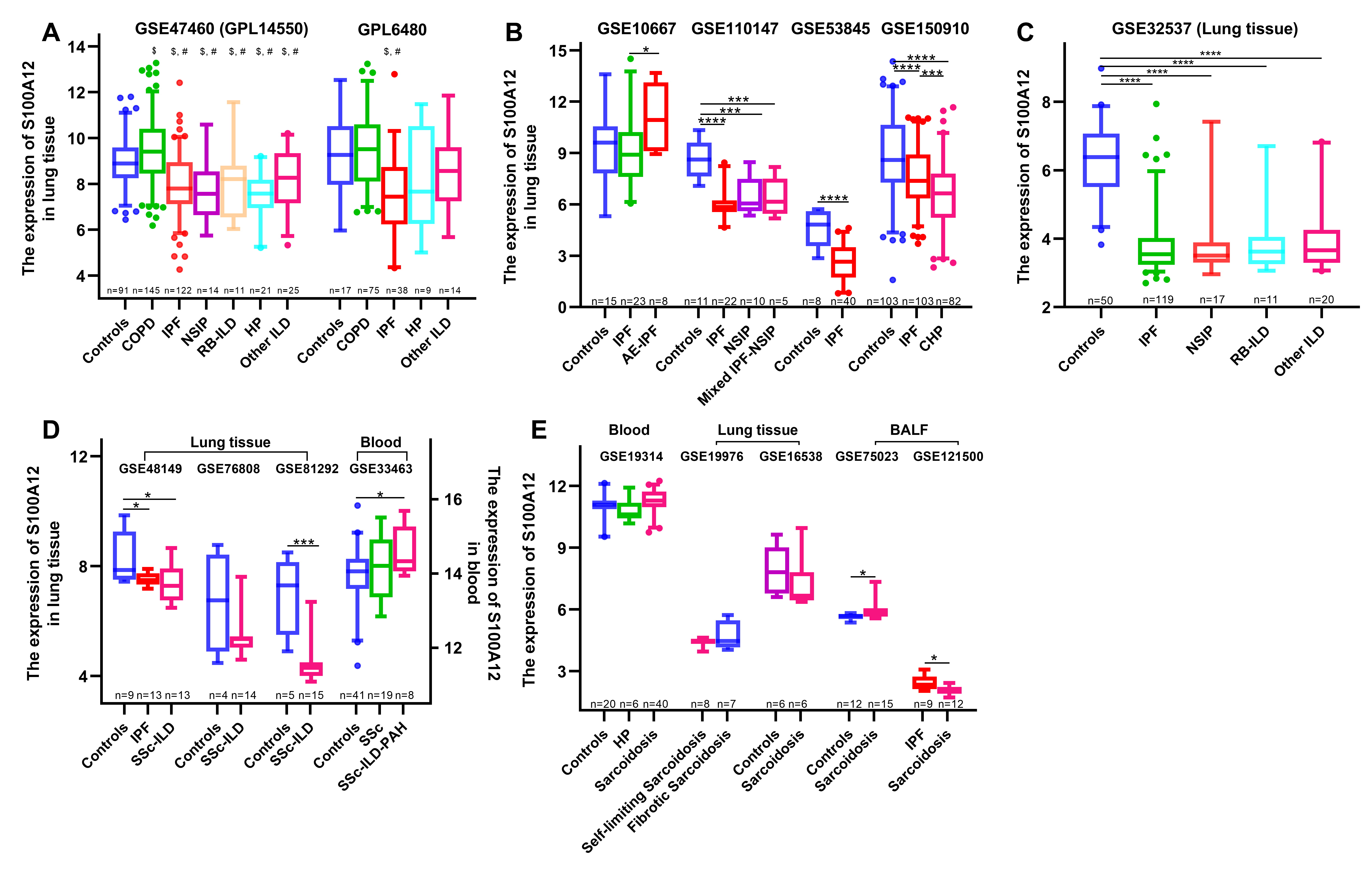

Supplement: Supplementary Figure 2 — Expression of S100A12 of lung tissue, blood and BALF. (A-C) The expression of S100A12 in human lung. (D) The expression of S100A12 in patients with SSc-ILD. (E) The expression of S100A12 in patients with sarcoidosis. $P < 0.05 vs. controls; #P < 0.05 vs. patients with COPD. P values were showed as: *P < 0.05; **P < 0.01; ***P < 0.001; ****P < 0.0001. COPD: chronic obstructive pulmonary disease; NSIP: non-specific interstitial pneumonia; HP: hypersensitivity pneumonitis; RB-ILD: respiratory bronchiolitis-related ILD; SSc-ILD: systemic sclerosis-related ILD; PAH: pulmonary arterial hypertension. [file Image_2.tif]

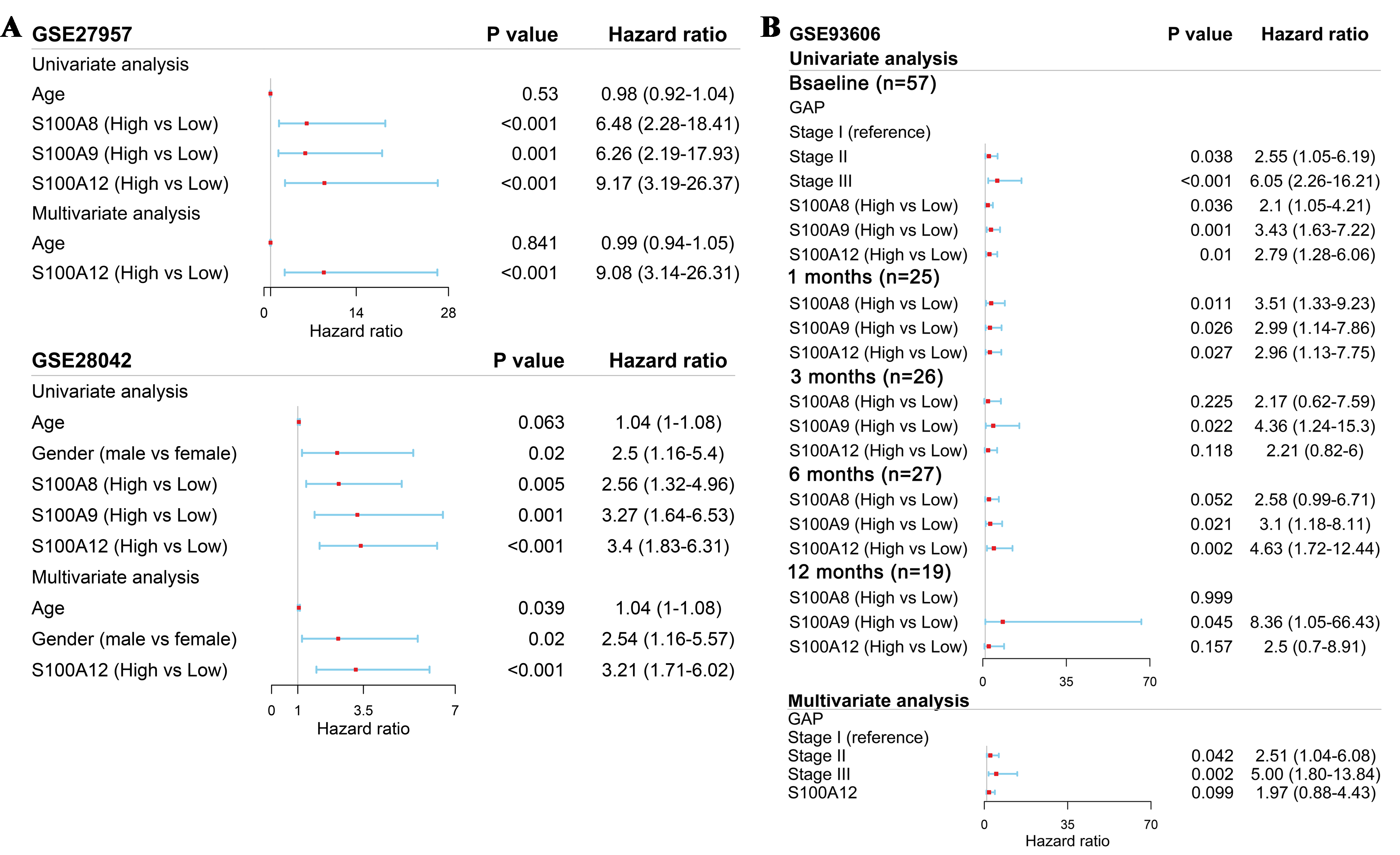

Supplement: Supplementary Figure 3 — Results of the cox regression regarding non-TFS in the GSE27957 and GSE28042 datasets (A), and the cox regression regarding non-PFS in the GSE93606 dataset (B). [file Image_3.tif]

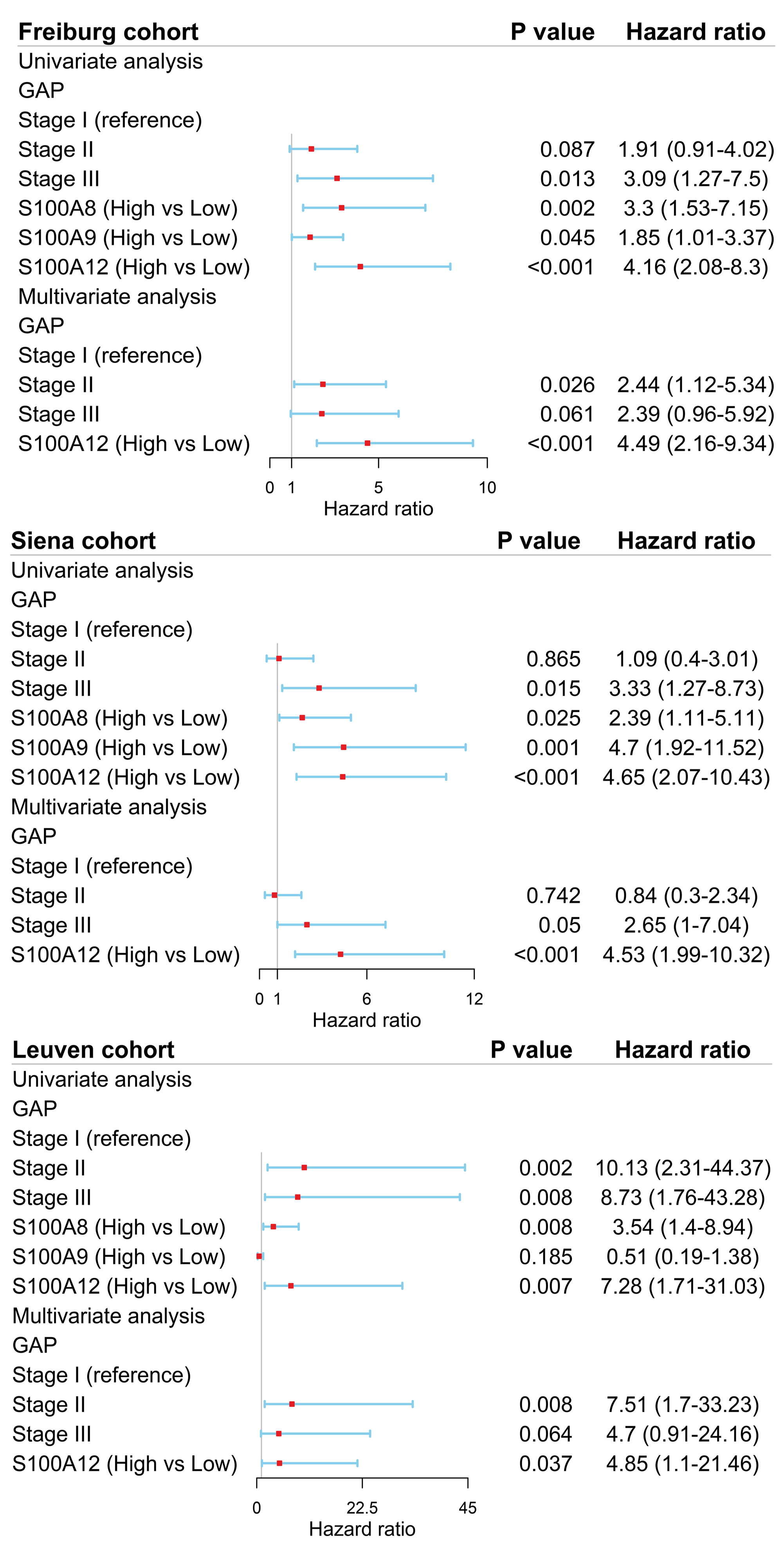

Supplement: Supplementary Figure 4 — Results of the cox regression regarding mortality in the GSE70866 dataset. [file Image_4.tif]

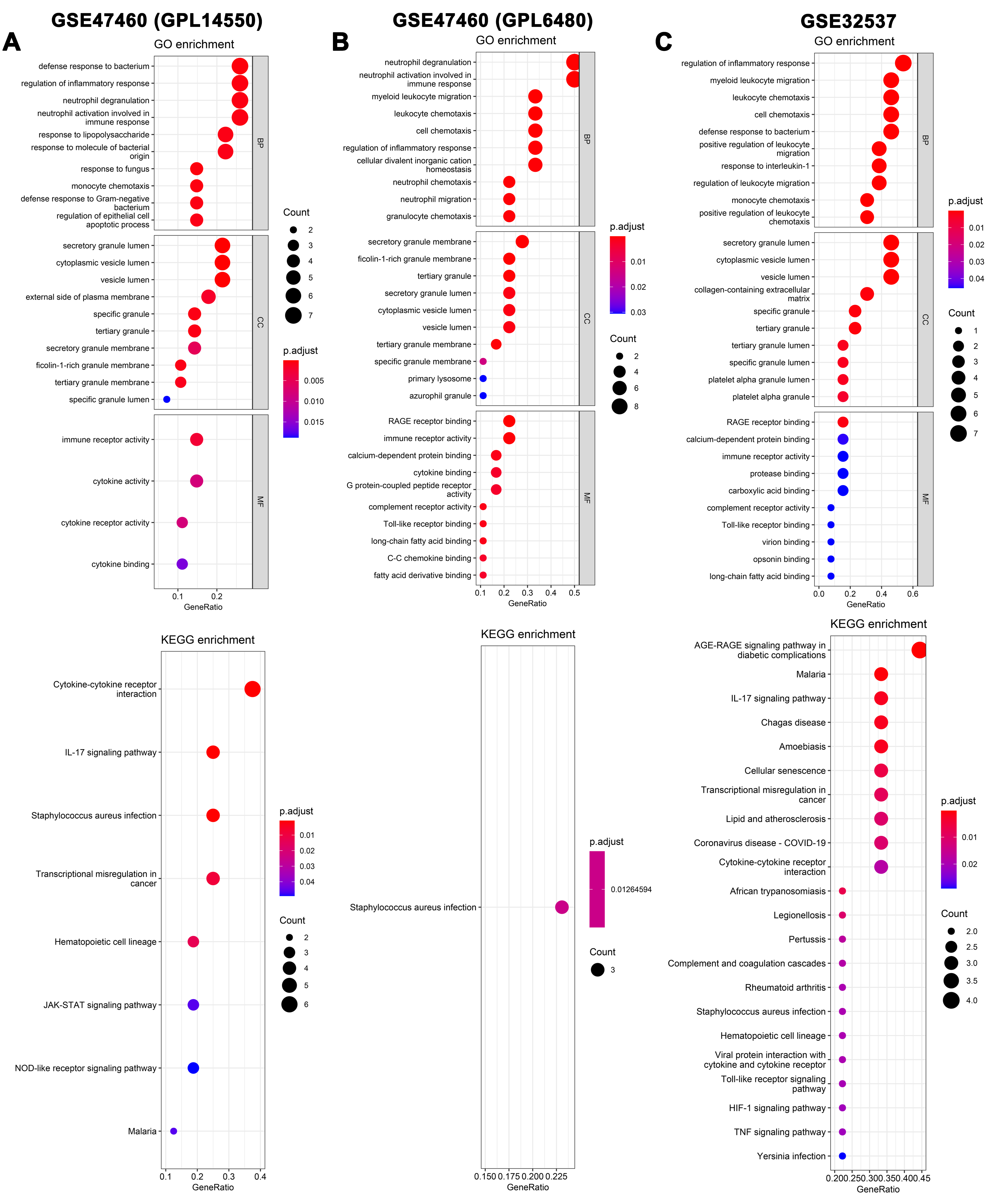

Supplement: Supplementary Figure 5 — Significant GO terms and KEGG pathways of DEGs between patients with high-expression and low-expression S100A12 (lung tissue). The top 10 significant terms for biological processes (BP), cellular component (CC), and molecular function (MF), and significant terms for KEGG pathways in the GSE47460 (GPL14550) (A), GSE47460 (GPL6480) (B), and GSE32537 datasets (C). [file Image_5.tif]

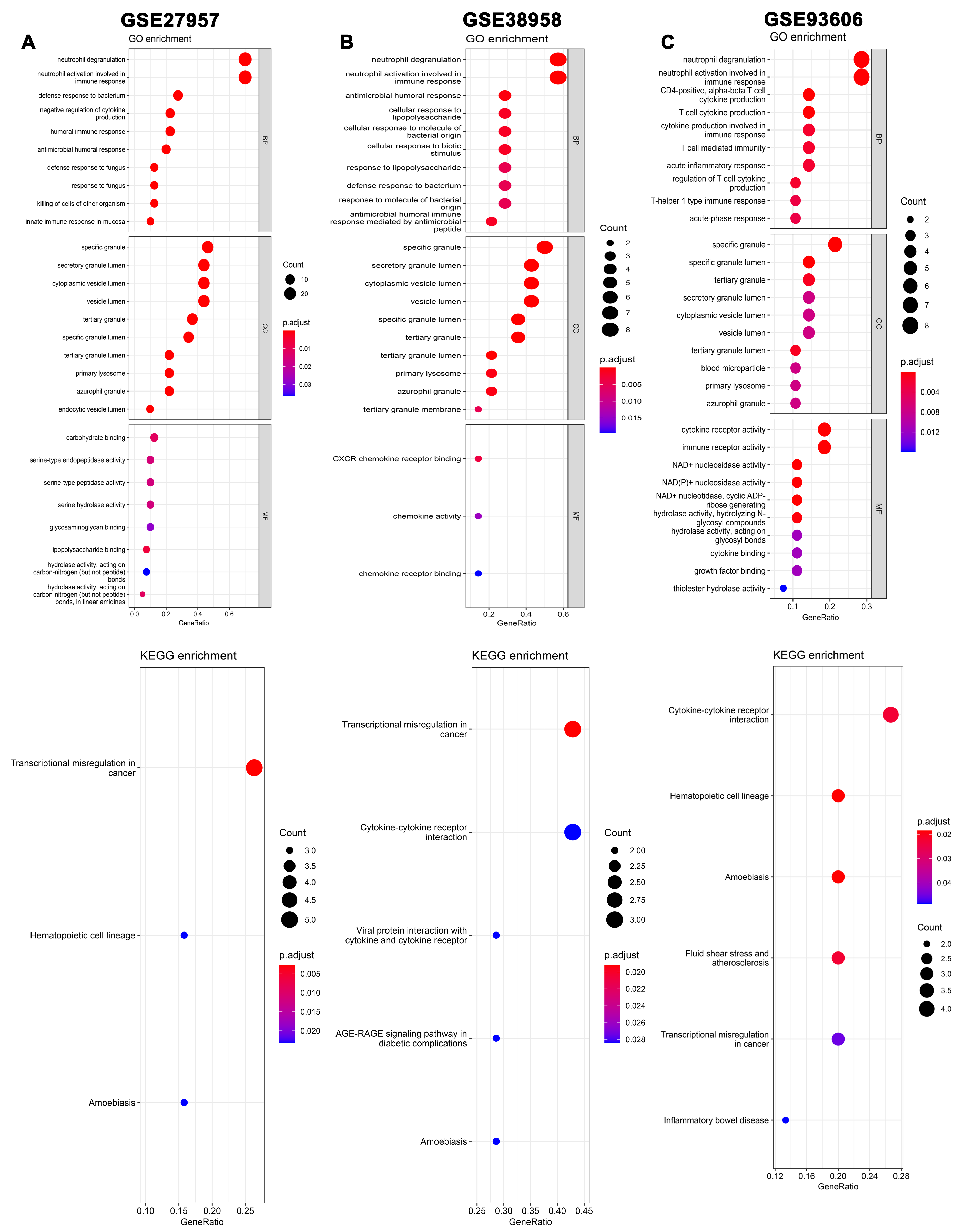

Supplement: Supplementary Figure 6 — Significant GO terms and KEGG pathways of DEGs between patients with high-expression and low-expression S100A12 (blood). The top 10 significant terms for biological processes (BP), cellular component (CC), and molecular function (MF), and significant terms for KEGG pathways in the GSE27957 (A), GSE38958 (B), and GSE93606 datasets (C). [file Image_6.tif]

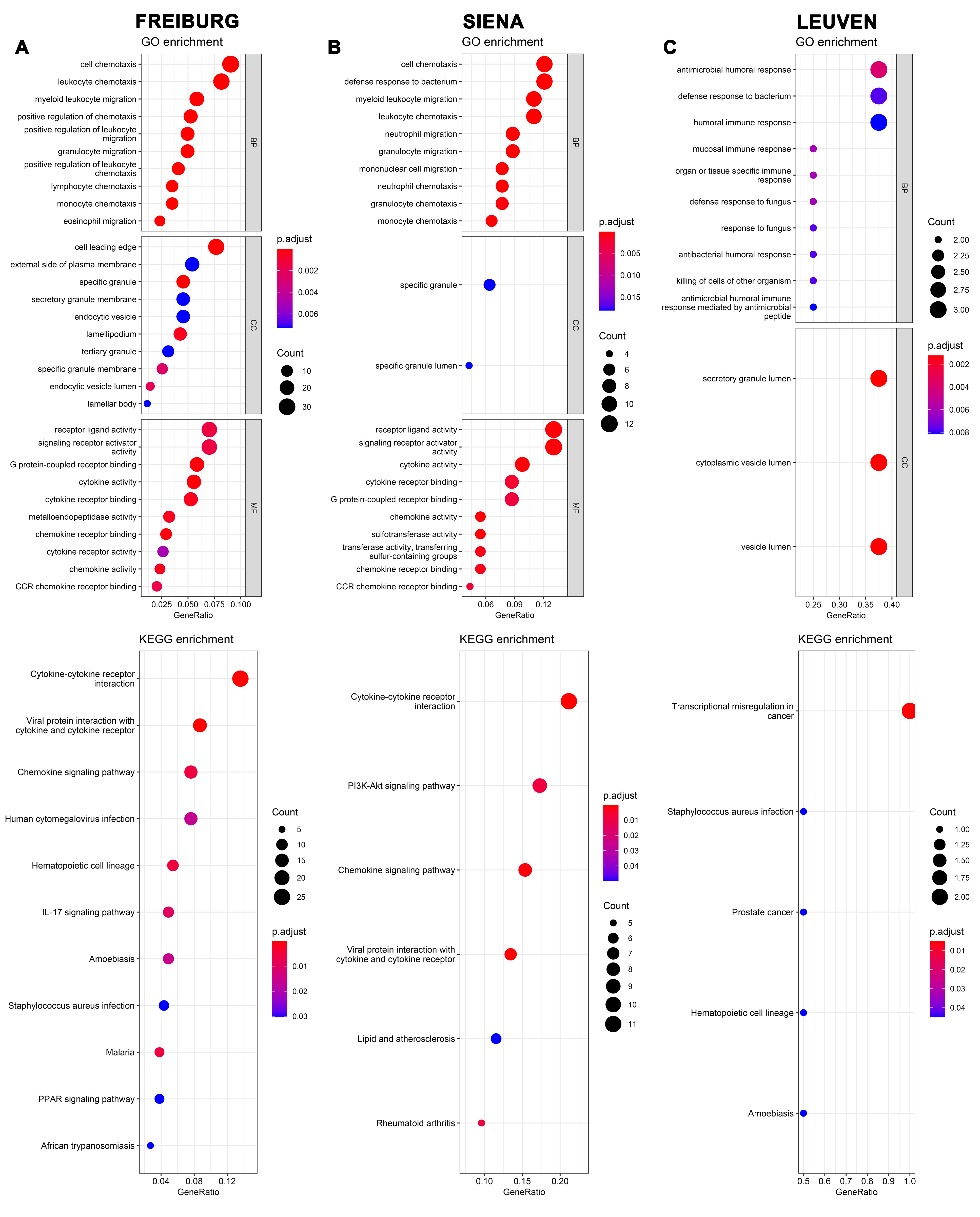

Supplement: Supplementary Figure 7 — Significant GO terms and KEGG pathways of DEGs between patients with high-expression and low-expression S100A12 according to GSE70866 dataset (BALF). The top 10 significant terms for biological processes (BP), cellular component (CC), and molecular function (MF), and significant terms for KEGG pathways in the FREIBURG cohort (A), SIENA cohort (B), and LEUVEN cohort (C). [file Image_7.tif]

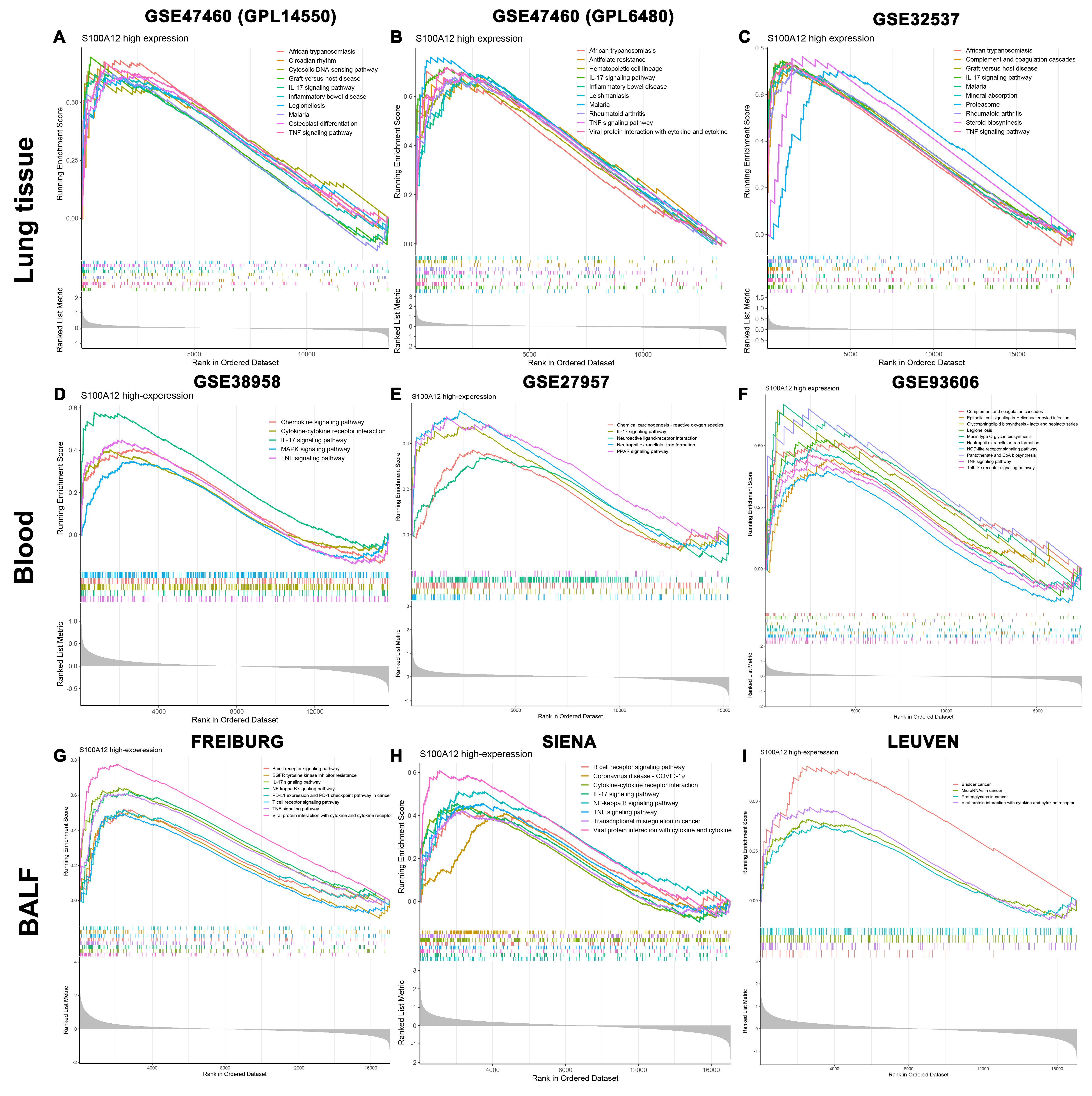

Supplement: Supplementary Figure 8 — GSEA analysis of DEGs between patients with high-expression and low-expression S100A12 in the three tissues. Lung tissue: (A) GSE47460 (GPL14550) dataset, (B) GSE47460 (GPL6480) dataset, (C) GSE32537 dataset. Blood: (D) GSE38958 dataset, (E) GSE27957 dataset, (F) GSE93606 dataset. BALF (GSE70866 dataset): (G) FREIBURG cohort, (H) SIENA cohort, (I) LEUVEN cohort. [file Image_8.tif]

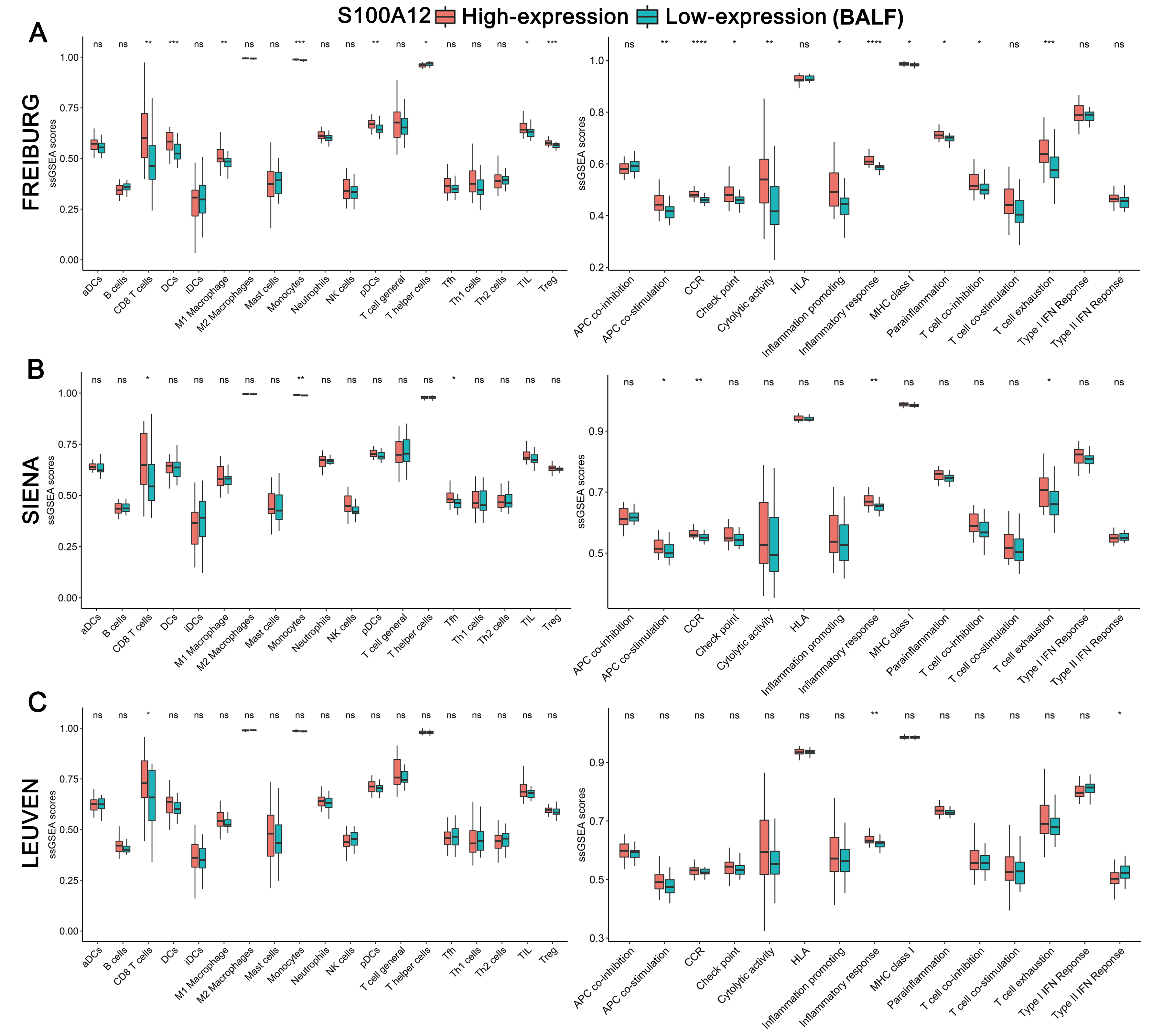

Supplement: Supplementary Figure 9 — Comparison of the BALF ssGSEA scores between patients with high-expression and low-expression S100A12 in the FREIBURG cohort (A), SIENA cohort (B), LEUVEN cohort (C) in the GSE70866 dataset. The scores of 19 immune cells are displayed in the left side, and 15 immune-related functions are displayed in the right side. DC, Dendritic Cell; TIL, Tumor infiltrates lymphocytes; CCR, cytokine-cytokine receptor. P values were showed as: ns, not significant; *P < 0.05; **P < 0.01; ***P < 0.001; ****P < 0.0001. [file Image_9.tif]

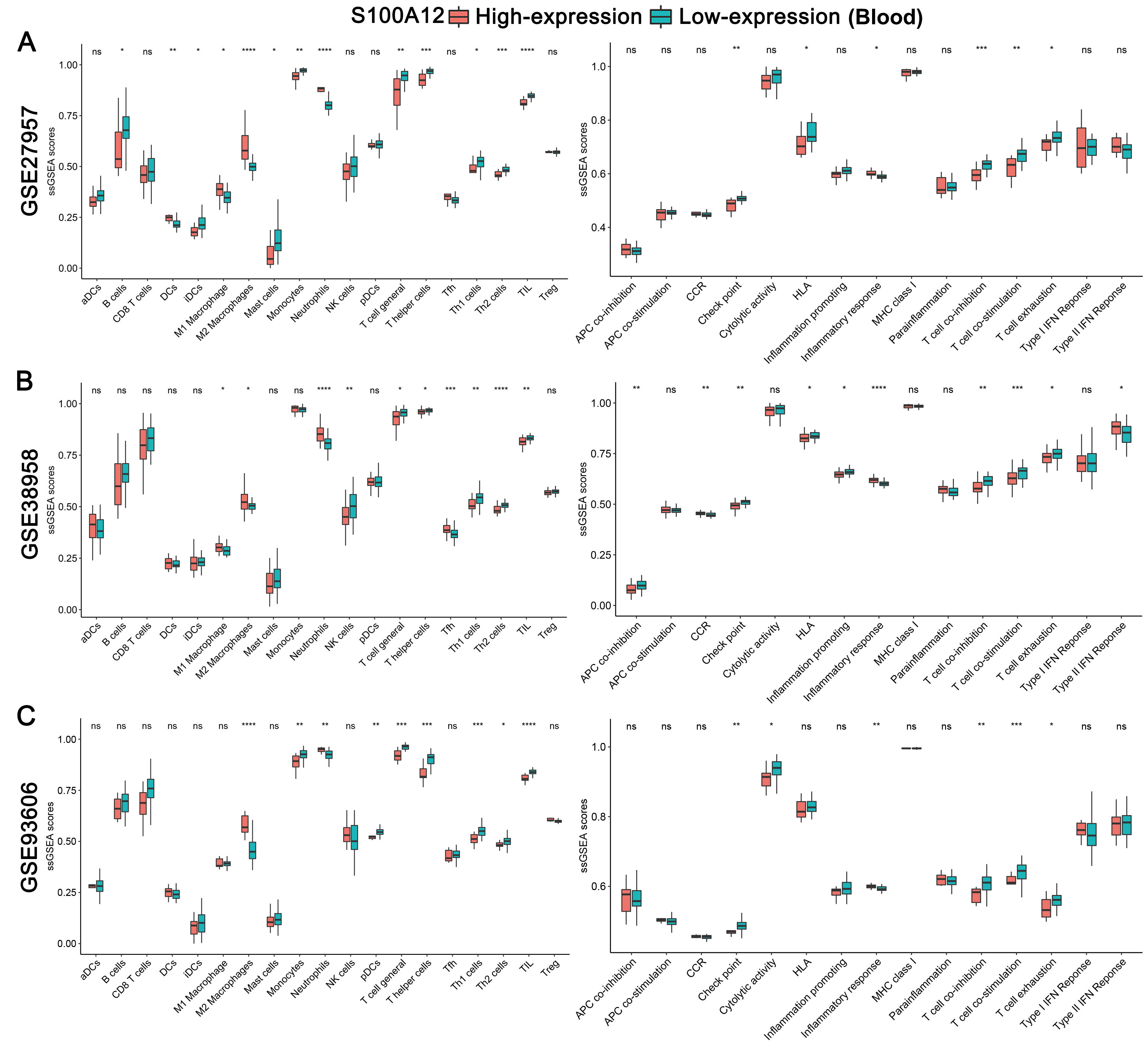

Supplement: Supplementary Figure 10 — Comparison of the blood ssGSEA scores between patients with high-expression and low-expression S100A12 in the GSE27957 (A), GSE38958 (B), and GSE93606 datasets (C). The scores of 19 immune cells are displayed in the left side, and 15 immune-related functions are displayed in the right side. DC, Dendritic Cell; TIL, Tumor infiltrates lymphocytes; CCR, cytokine-cytokine receptor. P values were showed as: ns, not significant; *P < 0.05; **P < 0.01; ***P < 0.001; ****P < 0.0001. [file Image_10.tif]

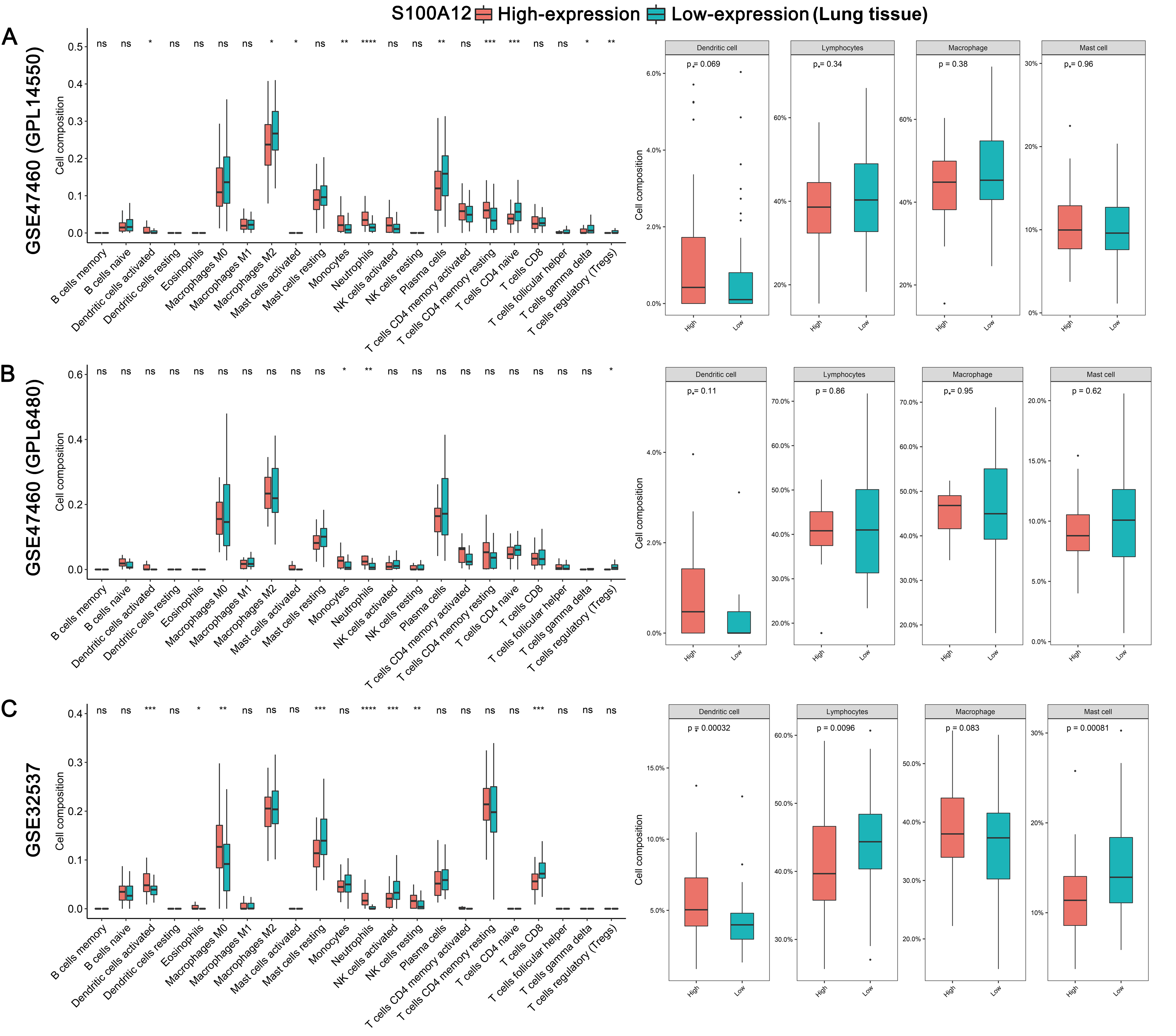

Supplement: Supplementary Figure 11 — Comparison of the lung CIBERSORT scores between patients with high-expression and low-expression S100A12 in the GSE47460 (GPL14550) (A), GSE47460 (GPL6480) (B), and GSE32537 datasets (C). The scores of 22 immune cells are displayed in the left side, and the scores of 4 composited cell types are displayed in the right side. P values were showed as: ns, not significant; *P < 0.05; **P < 0.01; ***P < 0.001; ****P < 0.0001. [file Image_11.tif]

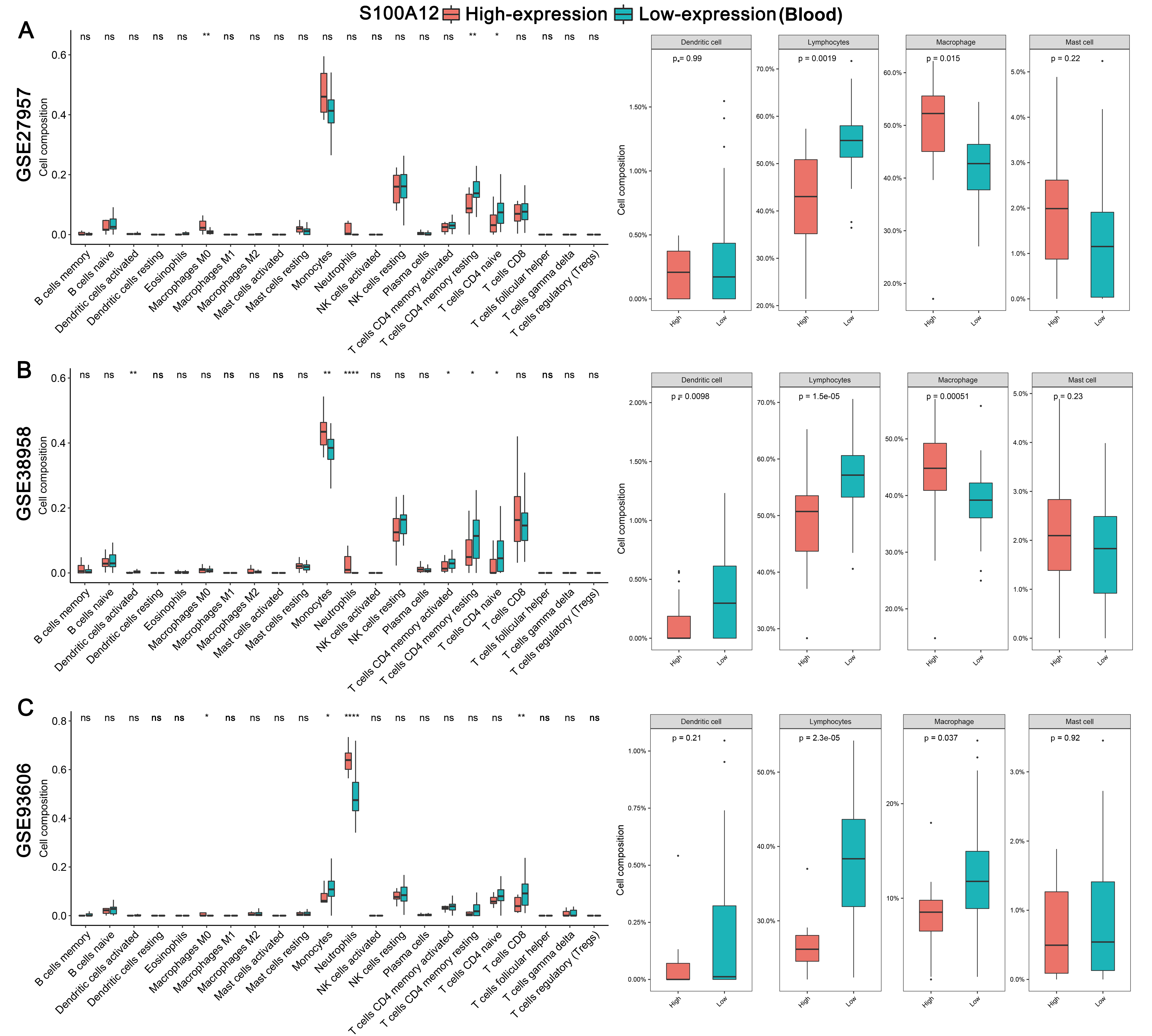

Supplement: Supplementary Figure 12 — Comparison of the blood CIBERSORT scores between patients with high-expression and low-expression S100A12 in the GSE27957 (A), GSE38958 (B), and GSE93606 datasets (C). The scores of 22 immune cells are displayed in the left side, and the scores of 4 composited cell types are displayed in the right side. P values were showed as: ns, not significant; *P < 0.05; **P < 0.01; ***P < 0.001; ****P < 0.0001. [file Image_12.tif]

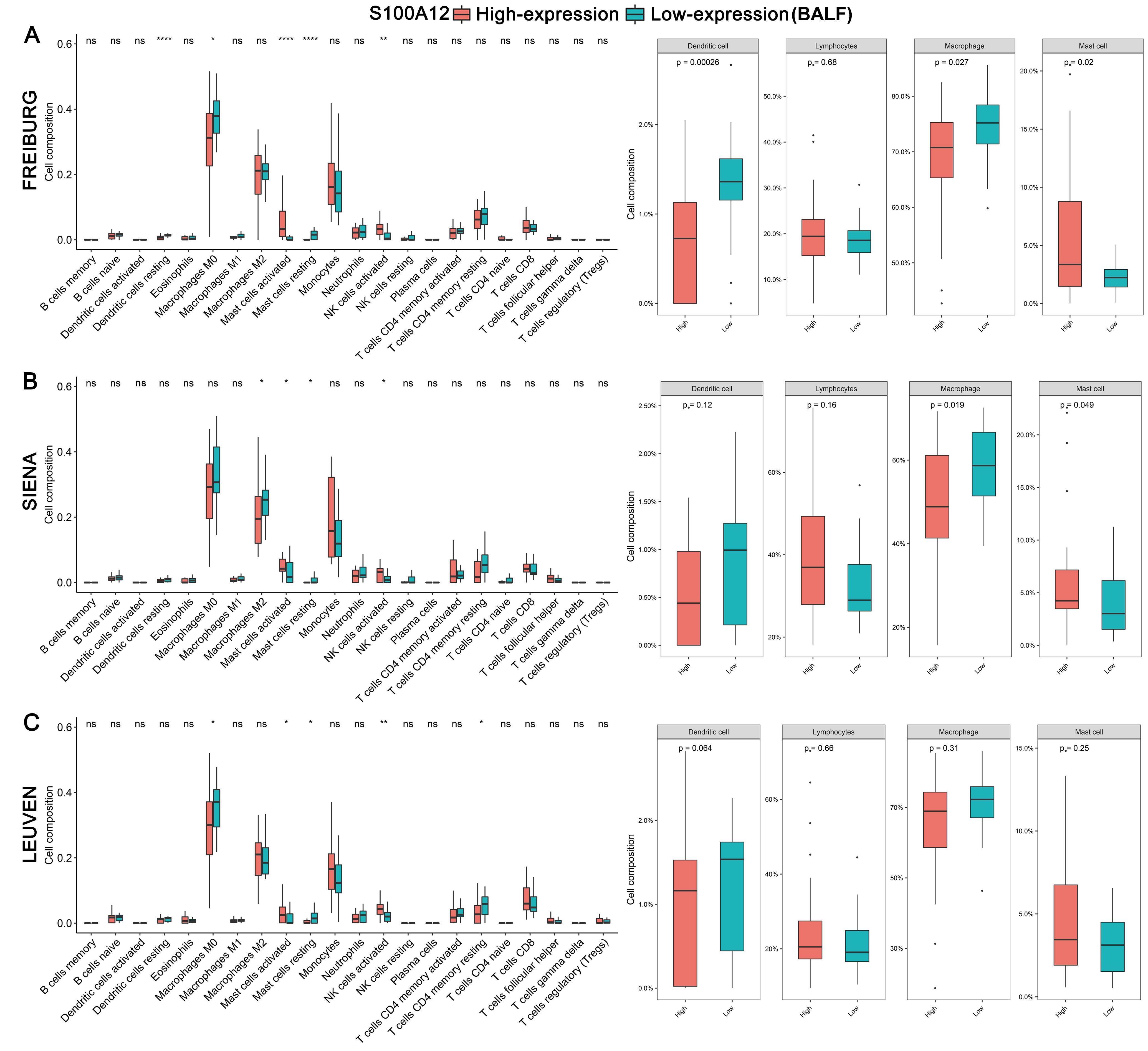

Supplement: Supplementary Figure 13 — Comparison of the BALF CIBERSORT scores between patients with high-expression and low-expression S100A12 in the FREIBURG cohort (A), SIENA cohort (B), LEUVEN cohort (C) in the GSE70866 dataset. The scores of 22 immune cells are displayed in the left side, and the scores of 4 composited cell types are displayed in the right side. P values were showed as: ns, not significant; *P < 0.05; **P < 0.01; ***P < 0.001; ****P < 0.0001. [file Image_13.tif]

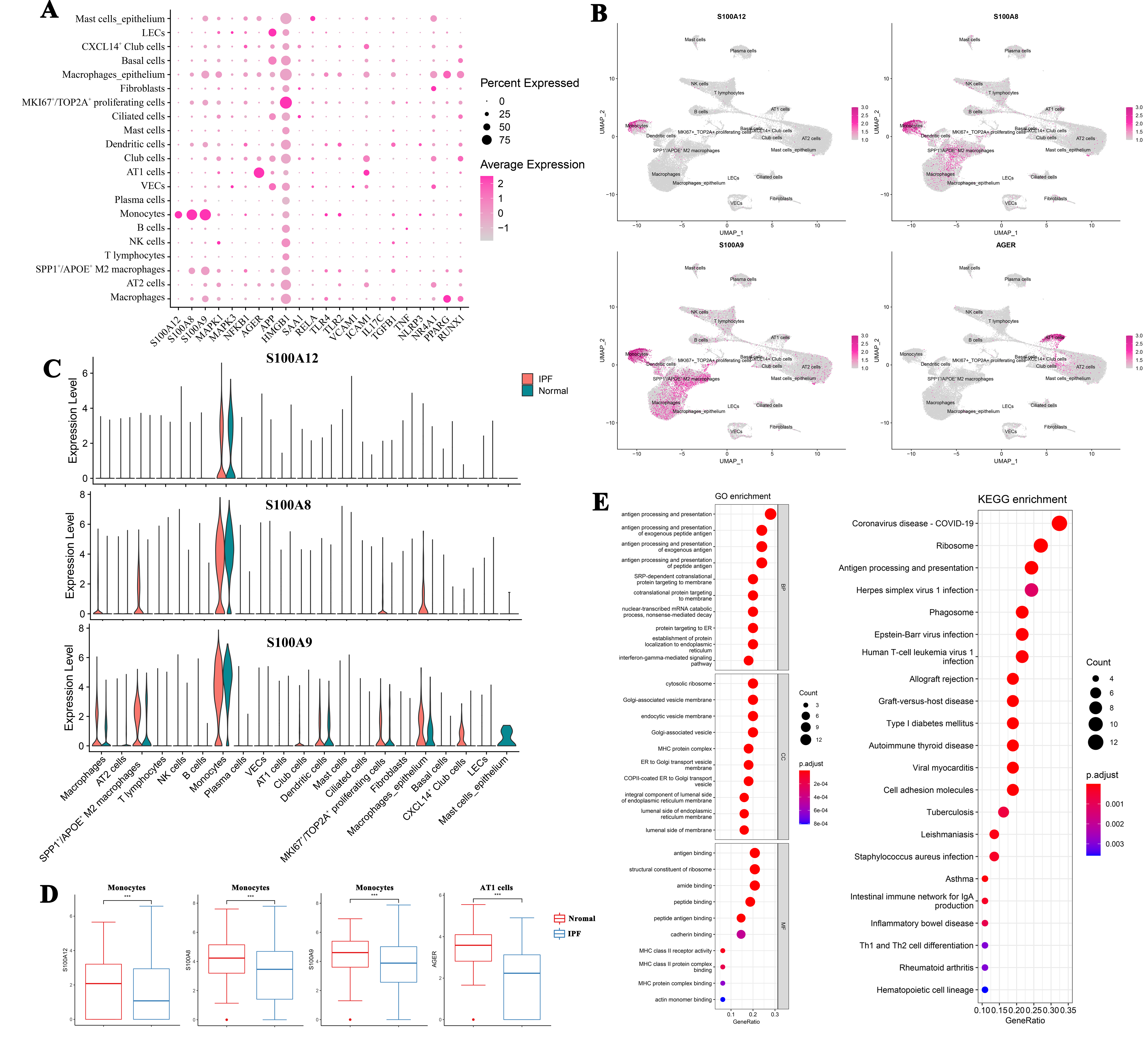

Supplement: Supplementary Figure 14 — The scRNA-seq analysis in the GSE122960 dataset (IPF = 4, control = 4). (A) Color dot plot of S100A12 and its partners; (B) Feature plot of S100A12, S100A8, S100A9, and AGER; (C) Violin plot of S100A12, S100A8, and S100A9. (D) The different expressive analysis of S100A12, S100A8, S100A9, and AGER between IPF controls in the selected cell; (E) The GO and KEGG analysis of DEGs between IPF and controls in the monocytes with S100A12 > 0. [file Image_14.tif]
